# Epidemiological and evolutionary analysis of canine circovirus from 1996 to 2023

**DOI:** 10.1186/s12917-024-04186-6

**Published:** 2024-07-20

**Authors:** Yumeng Liu, Yan Qin, Yanqing Hu, Wei Chen, Zhixiao Han, Chizhe Yi, Jingshan Bi, Haixin Huang, Yuying Li, XinYu Zhang, Tian Lan, Min Zheng, Wenchao Sun

**Affiliations:** 1 College of Animal Science and Technology, Guangxi University, Nanning, 530004 China; 2 Wenzhou Key Laboratory for Virology and Immunology, Institute of Virology, Wenzhou University, Wenzhou, 325035 China; 3 Guangxi Centre for Animal Disease Control and Prevention, Nanning, 530001 China

**Keywords:** Canine circovirus (CanineCV), Genetic diversity, Evolution, Origin, Transmission dynamics

## Abstract

**Background:**

Canine circovirus (CanineCV), a non-enveloped virus with a circular DNA genome, has been identified in various avian and mammalian species, including domestic and wild canids. This study aimed to comprehensively analyze the prevalence of CanineCV across diverse animal species in 11 provinces of China.

**Results:**

A total of 1,666 serum samples were collected, revealing a 5.82% prevalence of CanineCV in dogs, with the highest rates being observed in southern and eastern China. Phylogenetic analysis of 266 global CanineCV genomes sourced from the NCBI identified six distinct genotypes, elucidating the complex dynamics of their evolution. Evidence suggested a potential bat origin for CanineCV, with positive selection and high rates of evolution being observed. Recombination analysis revealed dynamic genetic exchange, highlighting the intricate nature of CanineCV evolution. Mutational analysis identified key amino acid substitutions likely to influence the virus’s adaptation. Additionally, glycosylation, palmitoylation, and SUMOylation sites were predicted, shedding light on crucial functional properties of the virus.

**Conclusions:**

This study provides a global perspective on the origin, genetic diversity, and evolutionary dynamics of CanineCV. Understanding these factors is crucial for elucidating its epidemiology and potential health risks.

**Supplementary Information:**

The online version contains supplementary material available at 10.1186/s12917-024-04186-6.

## Introduction

**Fig. 1:**
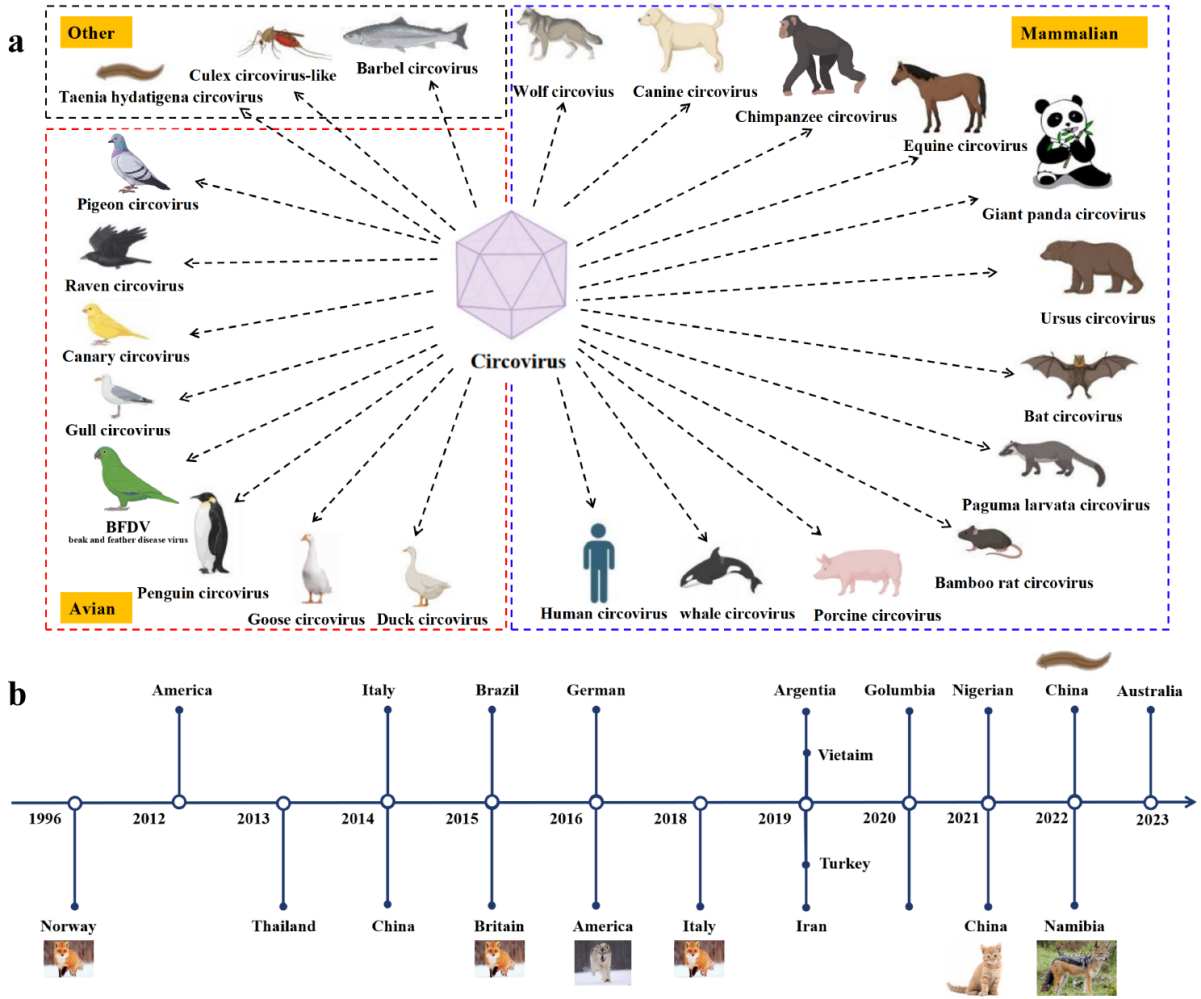
(**a**) Species taxonomy of circoviruses. (**b**) Regional timeline of canine circovirus discovery since 1996

Circoviruses have been identified in a wide range of species, particularly among birds and mammals. Hosts include parrot [1], goose [2], canary [3], gull [4], penguin [5], raven [6], duck [7], pigeon [8], bat [9], giant panda [10], *Paguma larvata* [11], chimpanzee [12], *Ursus americanu* [13], whale [14], equines [15], canines [16], human [12], pig [17], and bamboo rat [18]. Circoviruses have also been detected in organisms such as Taenia hydatigena [19], Culex mosquitoes [20], and barbel fish [21]. Notably, the Beak and Feather Disease Virus (BFDV)-also a member of the Circoviridae family-infects various avian species [22]. The taxonomy of circoviruses is illustrated in Fig. 1a.

Canine circovirus (CanineCV) is an icosahedral, non-enveloped virus with a single-stranded circular DNA genome of approximately 2 kb, classified within the family *Circoviridae* [23]. It was initially reported in serum samples from dogs in USA [16] and subsequently in Argentina [24], Brazil [25], Colombia [24], China [26, 27], Germany [28], Italy [29], Iran [30], and Thailand [31]. Australia has also reported cases and an indirect enzyme-linked immunosorbent assay based on virus-like particles has been developed [32]. CanineCV has also been detected in other animals, including wolves (*Canis lupus*) [33], badgers (*Meles meles*), foxes (*Vulpes vulpes*) [34], jackals (*Lupulella mesomelas*) [35], cats [36], and parasitic flatworms [37]. The timeline of CanineCV discovery from 1996 to the present is illustrated in Fig. 1b.

Dogs affected by CanineCV often experience co-infections with other pathogens that worsen their symptoms. For example, co-infection with Canine Parvovirus (CPV) has been linked to increased vomiting, diarrhea, and dehydration. CPV-induced immunosuppression and gastrointestinal damage create conditions that allow CanineCV to thrive, thereby increasing the severity of CanineCV infections [38]. Similarly, co-infection with Canine Distemper Virus (CDV) results in more pronounced symptoms, including fever, diarrhea, vomiting, loss of appetite, and respiratory distress. CDV can lead to more severe immunosuppression, increasing the severity of CanineCV symptoms and prolonging the course of the disease [39]. There have also been documented cases of the simultaneous presence of CanineCV, Canine Adenovirus types 1 and 2, and CPV [40]. Co-infections with other pathogens can exacerbate the symptoms of CanineCV in dogs via several mechanisms. Firstly, co-infecting pathogens can suppress the host immune system, making it easier for CanineCV to replicate and cause disease. For instance, infection with CDV or CPV can weaken the immune response, leading to more severe CanineCV symptoms. Secondly, co-infections may create a more favorable environment for CanineCV replication. Inflammation caused by other infections can increase the availability of cellular resources that CanineCV exploits for its own replication. Thirdly, co-infecting pathogens may help CanineCV evade the host immune system. Certain bacteria or viruses can interfere with the normal immune response, allowing CanineCV to persist longer in the host. Finally, co-infections can cause additional tissue damage, exacerbating the effects of CanineCV and leading to more severe clinical signs and a higher likelihood of complications.

The focus of this study was the prevalence of CanineCV among various animal species, including dogs, foxes, raccoons, and cats, in 11 provinces of China. The primary objectives were to gain a comprehensive understanding of the molecular genetic relationships associated with the virus and to provide a detailed insight into its genetic evolution. To achieve this, a meticulous analysis was conducted of all the available CanineCV sequences held in GenBank up to November 2023, employing both Maximum Likelihood (ML) and Bayesian Markov Chain Monte Carlo (MCMC) methods. The study gives a global perspective on critical aspects of CanineCV, such as its origin, genetic divergence, and evolutionary dynamics, and provides evidence of positive selection and high rates of evolution that increase our knowledge of the virus’s properties and characteristics.

## Materials and methods

### Epidemiological sample collection and sequencing

An extensive surveillance initiative was undertaken in China from 2017 to 2019 to monitor the presence of rabies antibodies. The study involved several species, including domestic pets such as dogs and cats, rural dogs, and wild animals such as foxes and raccoons. Serum samples were collected from pets diagnosed in veterinary hospitals and rural dogs undergoing rabies antibody monitoring at the Guangxi Animal Disease Prevention and Control Center, China. Additionally, feces samples were collected from foxes and raccoons in Liaoning Province (northeastern China) with the cooperation of veterinary diagnostic institutions.

Polymerase Chain Reaction (PCR) analysis was used to identify the presence of rabies antibodies and CanineCV viruses. Positive samples were identified based on specific PCR amplification of target sequences associated with these pathogens. Samples with positive CanineCV PCR results were selected for sequencing. Those with the highest quality and concentration of viral DNA were selected to ensure accurate and complete genome assembly. Subsequent assembly and editing of viral sequences were carried out using BioEdit software.

### Recombination analysis

Recombination analysis was performed using RDP4 software [41]. Complete genome sequences of CanineCV-positive samples were aligned using MEGA v.7. Various algorithms within RDP4, including RDP, GENECONV, BootScan, MaxChi, Chimaera, SiScan, and 3Seq, were used to detect potential recombination events. Recombination events were considered valid only if identified by more than five methods with a p-value lower than 0.05 after Bonferroni correction. Characterization of potential recombinant strains was carried out to understand the evolutionary dynamics of CanineCV.

### Phylogenetic analysis

Phylogenetic analysis tracing the bat origin of CanineCV used Maximum Likelihood (ML) tree reconstruction with Rep gene sequences from CanineCV and related circoviruses, including bat variants. These sequences were aligned using MEGA v.7 and selected from GenBank for their relevance. The General Time Reversible (GTR) model with empirical base frequencies and a three-category Gamma distribution (GTR + G + I) was chosen as the best fit based on the lowest Bayesian Information Criterion (BIC) score, determined by ModelFinder in the PhyloSuite program.

**Fig. 2:**
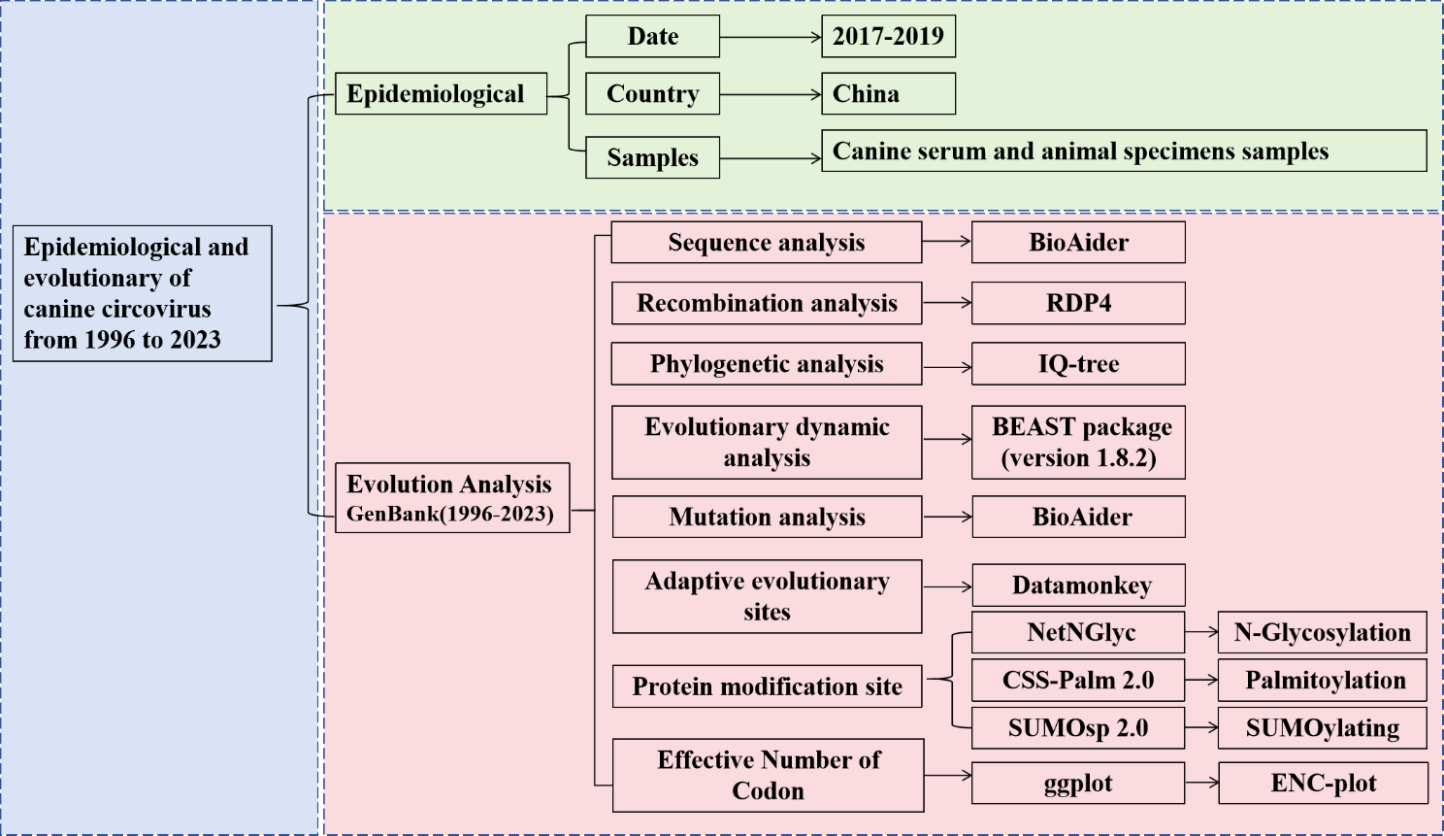
Flow chart of the epidemiological and evolutionary analysis of canine circovirus

A focused analysis of CanineCV from 1996 to 2023 also encompassed over 275 globally sourced canine sequences originating from various regions, including Africa, Australia, East Asia, Europe, the Middle East, North America, South America, South Asia, and Southeast Asia. A further 64 genomes from the present study were integrated into this dataset. The flow chart in Fig. 2 illustrates the epidemiological and evolutionary analyses of CanineCV undertaken in this study.

Phylogenetic tree reconstruction was based on complete genome sequences, with the substitution model (GTR + G4 + I) being chosen on the basis of the lowest BIC score in PhyloSuite [42]. The reliability of inferred clades was assessed using 1000 bootstrap replicates. Detection of potential recombination breakpoints was achieved using the GARD algorithm, while the RDP4 program was used to characterize potential recombinant strains. Recombination events were considered valid only if identified by more than two methods with a p-value lower than 0.001 after Bonferroni correction.

### Evolutionary dynamics analysis

A serial coalescent analysis was performed on the complete genomes of non-recombinant CanineCV strains. The Bayesian framework in BEAST v.1.10.4 [43] was used, selecting the GTR + G4 + I substitution model as the best fit based on the lowest BIC score as determined by ModelFinder in PhyloSuite. Log-normal relaxed molecular clock and skyline population dynamics models were chosen. Two MCMC [44] runs of 100 million generations were executed, and parameters and trees were sampled every ten thousand generations. Posterior distributions and convergence were assessed using Tracer v.1.7, summarizing parameter estimations in terms of mean and 95% highest posterior density.

### Amino acid mutations analysis

Pairs of continuously coding genes were combined in tandem sequence in BioAider and used as reference sequences for analysis of genome variation within CanineCV. The corresponding viral sequences that contained these possible linkage substitution sites were subsequently extracted using BioAider.

### Functional analysis of the adaptive evolutionary sites of proteins in CanineCV

To identify sequences on the *Rep* gene, an ML tree based on the available sequences was reconstructed using DataMonkey (http://www.datamonkey.org/). The methods used to identify amino acid positions were: fixed effects likelihood (FEL), single-likelihood ancestor counting (SLAC), mixed effects model of evolution (MEME), and fast unconstrained Bayesian approximation (FUBAR) [45–47].

### Prediction of glycosylation, palmitoylation, and SUMOylation sites

The NetNGlyc web server was used to predict N-glycosylation sites in the *Rep* protein [36]. NPS/T sequences were excluded from the analysis and only values above a predefined cut-off of 0.5 were considered positive for potential glycosylation. CSS-Palm v.2.0 and SUMOsp v.2.0 programs were employed to determine palmitoylation/acylation sites and SUMOylation sites, respectively [37].

### ENC plot analysis of codon usage bias

An effective number of codons (ENC) value is an absolute statistic for evaluating the decisive factors shaping codon usage bias. Values were calculated for the GC content in synonymous codons at the third position (GC3s) [48]. If codon usage is limited only by G + C mutational bias, the expected ENC values will be close to, or on, the standard curve. Effective number of codons (ENC) values were calculated as follows:

$$ENC - {\rm{plot}} = 2 + S\left( {{{29} \over {{S^2} + {{(1 + S)}^2}}}} \right)$$

where S is the GC content at the third codon position, namely, GC3s.

## Results

### CanineCV detected in China

**Fig. 3:**
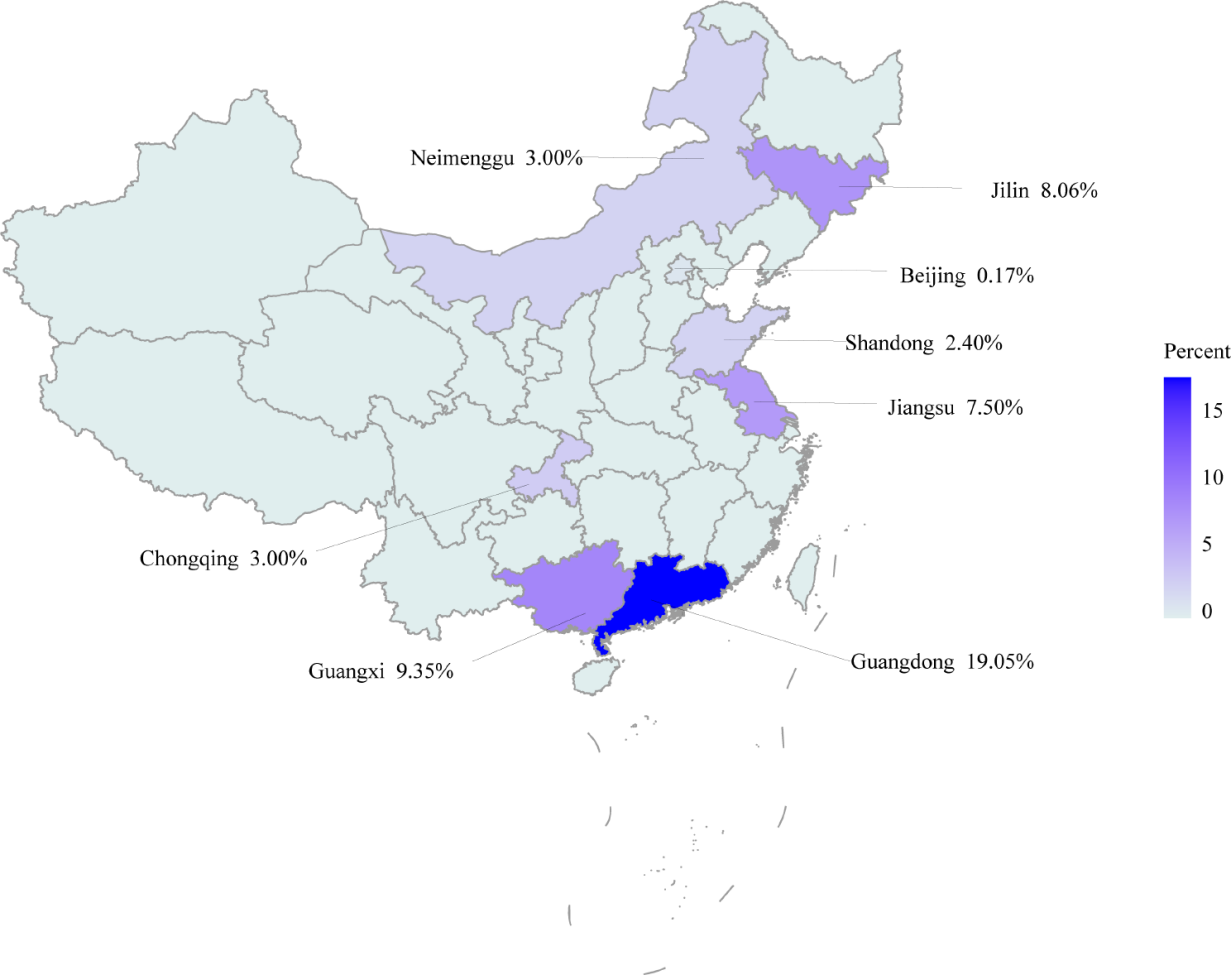
Geographical distribution of dogs in China testing positive for CanineCV DNA in blood serum by PCR (based on epidemiological data generated in this study)

Comprehensive monitoring of rabies antibodies in China from 2017 to 2019 resulted in collection of 1,666 serum samples from dogs. PCR analysis detected CanineCV DNA in 97 serum samples, giving a prevalence rate among dogs of 5.82% (Table 1). No positive cases were detected in samples from other species, giving a prevalence rate of 0%. Positive rates were relatively high in dogs in Guangdong (4/21) and Guangxi (72/770), with prevalence rates of 19.05% and 9.35%, respectively. By contrast, no positive cases were detected in Shanghai (0/31) and Sichuan (0/126), possibly due to the limited sample sizes in these provinces. The geographical distribution of CanineCV-positive cases is shown in Fig. 3. Positive rates among dogs were higher in southern and eastern China than in northern and western regions. Complete genomes of 64 CanineCV-positive dogs were successfully amplified (see Supplementary Table A1).

**Table 1:** Identifying information and CanineCV prevalence in samples screened for rabies antibodies in China from 2017 to 2019

Province	County	Year	Host	Sample type	Samples (*n*)	CanineCV positives	CanineCV positive rate (%)
Beijing	Beijing	2019	Urban pet dog, sick	serum	140	1	0.71
Chongqing	Chongqing	2019	Urban pet dog, sick	serum	100	3	3.00
Guangdong	Guangzhou	2019	Urban pet dog, sick	serum	21	4	19.05
Guangxi	Baise	2017	Rural dog, healthy	serum	104	16	15.38
	Baise	2018	Rural dog, healthy	serum	90	12	13.33
	Guigang	2019	Rural dog, healthy	serum	89	16	17.97
	Guilin	2017	Rural dog, healthy	serum	85	6	7.06
	Hechi	2017	Rural dog, healthy	serum	67	0	0.00
	Hechi	2018	Rural dog, healthy	serum	10	0	0.00
	Nanning	2017	Rural dog, healthy	serum	96	1	1.04
	Nanning	2018	Rural dog, healthy	serum	68	19	27.94
	Nanning	2019	Urban pet dog, sick	serum	22	1	4.55
	Qinzhou	2017	Rural dog, healthy	serum	99	1	1.01
	Yulin	2017	Rural dog, healthy	serum	40	0	0.00
	Nanning	2019	Urban pet cat, sick	feces	38	0	0.00
Jiangsu	Kunshan	2019	Urban pet dog, sick	serum	40	3	7.50
Jilin	Changchun	2019	Urban pet dog, sick	serum	62	5	8.06
Liaoning	Shenyang	2019	Domesticated foxes in fur farm	feces	37	0	0.00
	Shenyang	2019	Domesticated raccoons in fur farm	feces	13	0	0.00
Neimenggu	Huhehaote	2019	Urban pet dog, sick	serum	126	3	2.38
Shandong	Jinan	2019	Urban pet dog, sick	serum	250	6	2.40
Shanghai	Shanghai	2019	Urban pet dog, sick	serum	31	0	0.00
Sichuan	Chengdu	2019	Urban pet dog, sick	serum	126	0	0.00

### Evidence for the origin of emergent CanineCVs

**Fig. 4:**
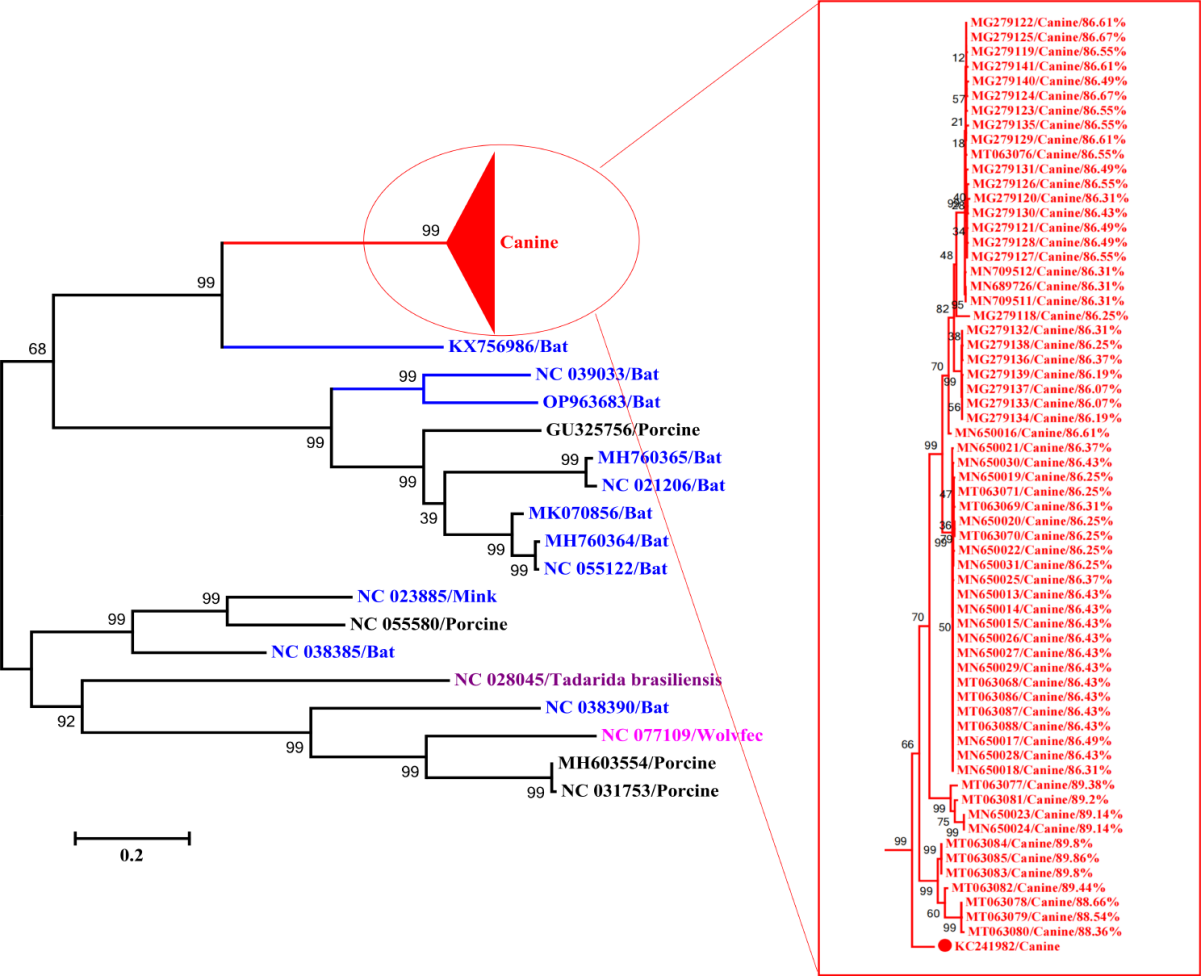
Phylogenetic tree reconstructed using the Maximum Likelihood method in IQ-TREE, based on 81 replication-associated protein genes (*Rep*) from various circoviruses. The evolutionary origin of CanineCV is inferred from the conserved coding regions of ORF1. Branches are annotated with posterior probability values and distinct clades are indicated by different colors. Percentages indicate sequence similarity and red dots mark the reference sequences

An ML tree was reconstructed using Rep gene sequences to trace the origin of CanineCV (Table A2 includes detailed gene sequences of the circovirus strains). All the CanineCV strains were closely related to the clade 1 bat circoviruses that had been isolated in China from 2011 to 2016 (Fig. 4). The circovirus 1 bat virus KX756986 (GenBank) formed an outgroup of the clade 1 bat CVs along with the CanineCV strains, suggesting they have a bat CV origin. It has previously been reported that CanineCVs are closely related to PCV3 strains when complete genomes are compared, and that PCV3s are closely related to bat strains, which supports the view that CanineCV may have originated from bat CV.

### Phylogenetic analysis and evolutionary dynamics of CanineCV

**Fig. 5:**
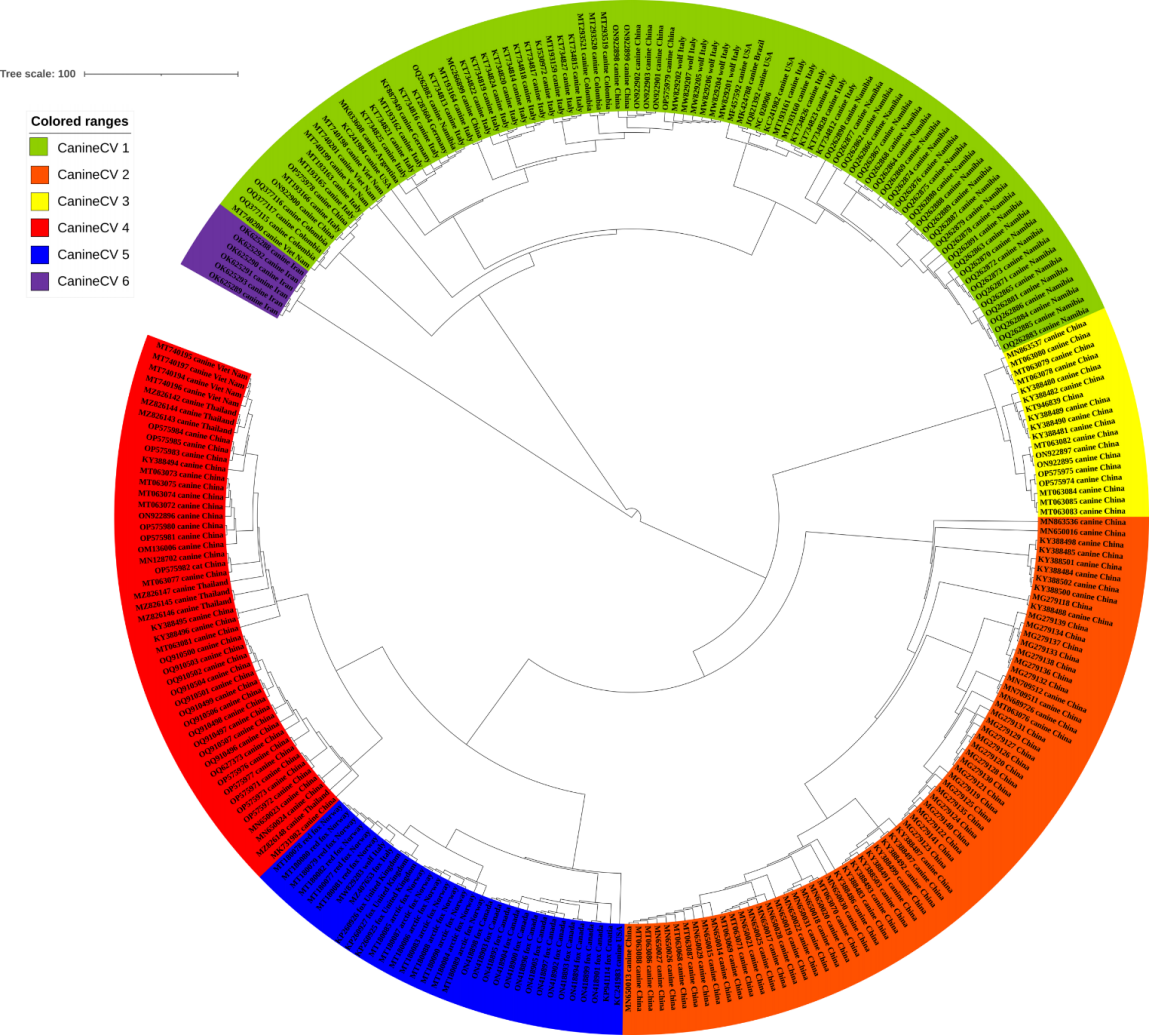
Maximum Likelihood phylogenetic tree of CanineCV evolution based on complete nucleotide sequences

**Fig. 6:**
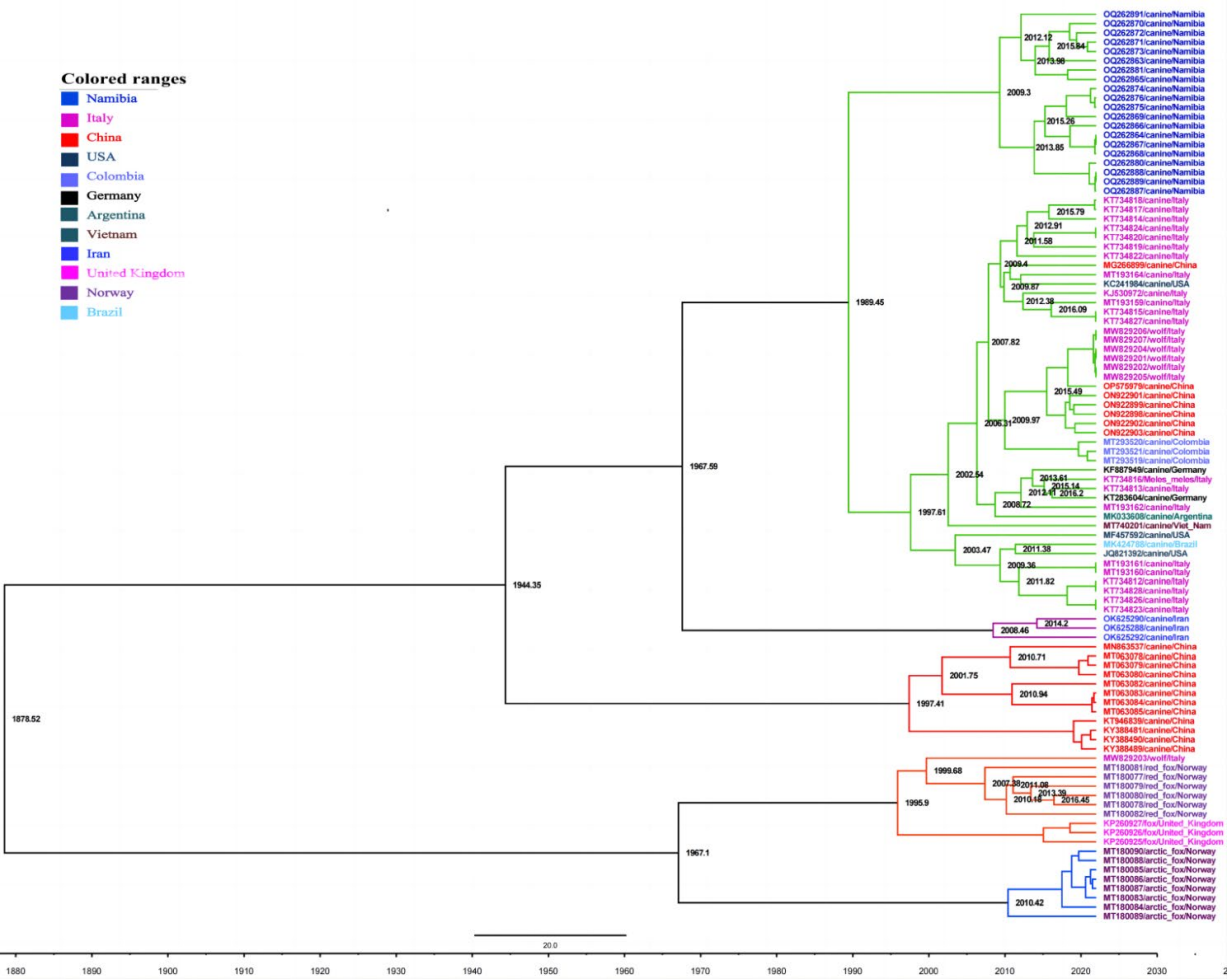
Maximum clade credibility tree reconstructed using BEAST (v.1.8.4) with 98 of the 266 CanineCV strains. The origins were deduced using the conserved coding region of complete coding sequences. The posterior is displayed along each branch and the various clades are indicated by different colors

Comprehensive analysis of 266 whole genome sequences obtained from NCBI (Table A3 includes detailed gene sequences of the circovirus strains) involved multiple sequence alignments and phylogenetic analyses and revealed the evolution of CanineCV into six distinct genetic groups, as shown in the whole genome ML tree (Fig. 5). CanineCV-1 was initially identified in Europe (specifically Italy) in 2010, and its subsequent spread to other European countries by 2012 is detailed in Fig. 6 [24]. Additional spatiotemporal insight into the distribution of CanineCVs within each genotype is available in Supplementary Table A4. Notably, CanineCV-1 was also detected in North America between 2011 and 2014, while the most recent discovery in dog serum was in Harbin, China. The global presence of CanineCV-1 in various species, including dogs, wolves, and badgers, highlights its widespread distribution [16]. CanineCV-2 was identified exclusively in China in 2014 and 2018, while CanineCV-3 and CanineCV-4 were found predominantly in China and certain Southeast Asian countries between 2011 and 2019. CanineCV-5 was more broadly distributed, being detected in numerous European countries from 1996 to 2017, with a few cases in Arctic and Norwegian red foxes [49]. The emergence of CanineCV-6 in Iran in 2019 created a distinct clade (Group 6 in Fig. 5) [30, 31]. The time (year) of the most recent common ancestor (tMRCA) for CanineCV was estimated to be 1878.52.

### Recombination analysis

Thirty-one potential recombination events were identified using RDP4 software, with recombination fragment sizes ranging from 7.08 to 87.78% of the genome (Table 2). Notably, these events occurred not only within a single ORF gene, but also involved two genomes and various other genomic regions. Events 1–17 involve recombination within the same genotype, while events 18–31 involve recombination between different genotypes. Intriguingly, the primary parents of these reorganization events were traced back to Canadian foxes and dogs from China and Italy. The secondary parents were identified in dogs from China, Italy, and Namibia. These events span diverse geographic locations, encompassing Namibia, Brazil, China, Italy, Colombia, Vietnam, Iran, the USA, Germany, and more. An understanding of these events is crucial for deciphering the genetic diversity and evolution of canine populations, and it suggests a dynamic exchange of genetic material between various regional populations. These findings shed light on the intricate nature of recombination events in dog and wolf populations across diverse regions, providing valuable insight into the dynamics of genetic evolution in these species.

**Table 2:**
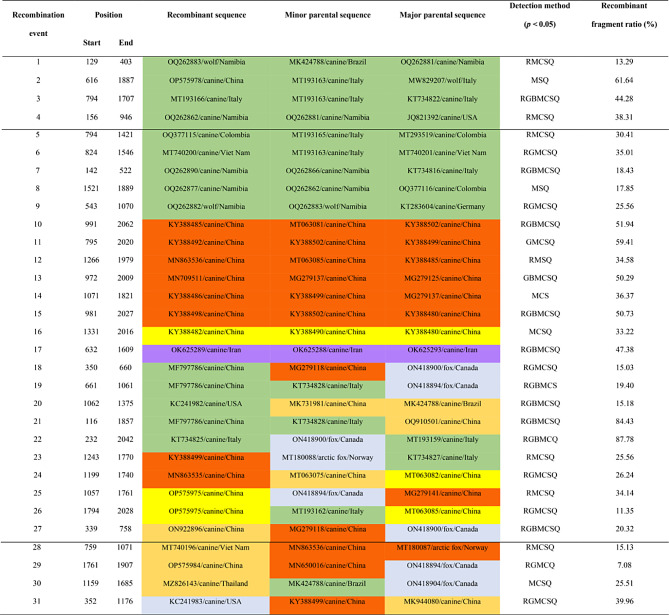
Potential recombination events within the same genotype (events 1–17) and between different genotypes (events 18–31) of canine circovirus color-coded as follows: CanineCV-1 green, CanineCV-2 orange, CanineCV-3 yellow, CanineCV-4 light yellow, CanineCV-5 light blue, and CanineCV-6 purple

### Mutation analysis

**Fig. 7:**
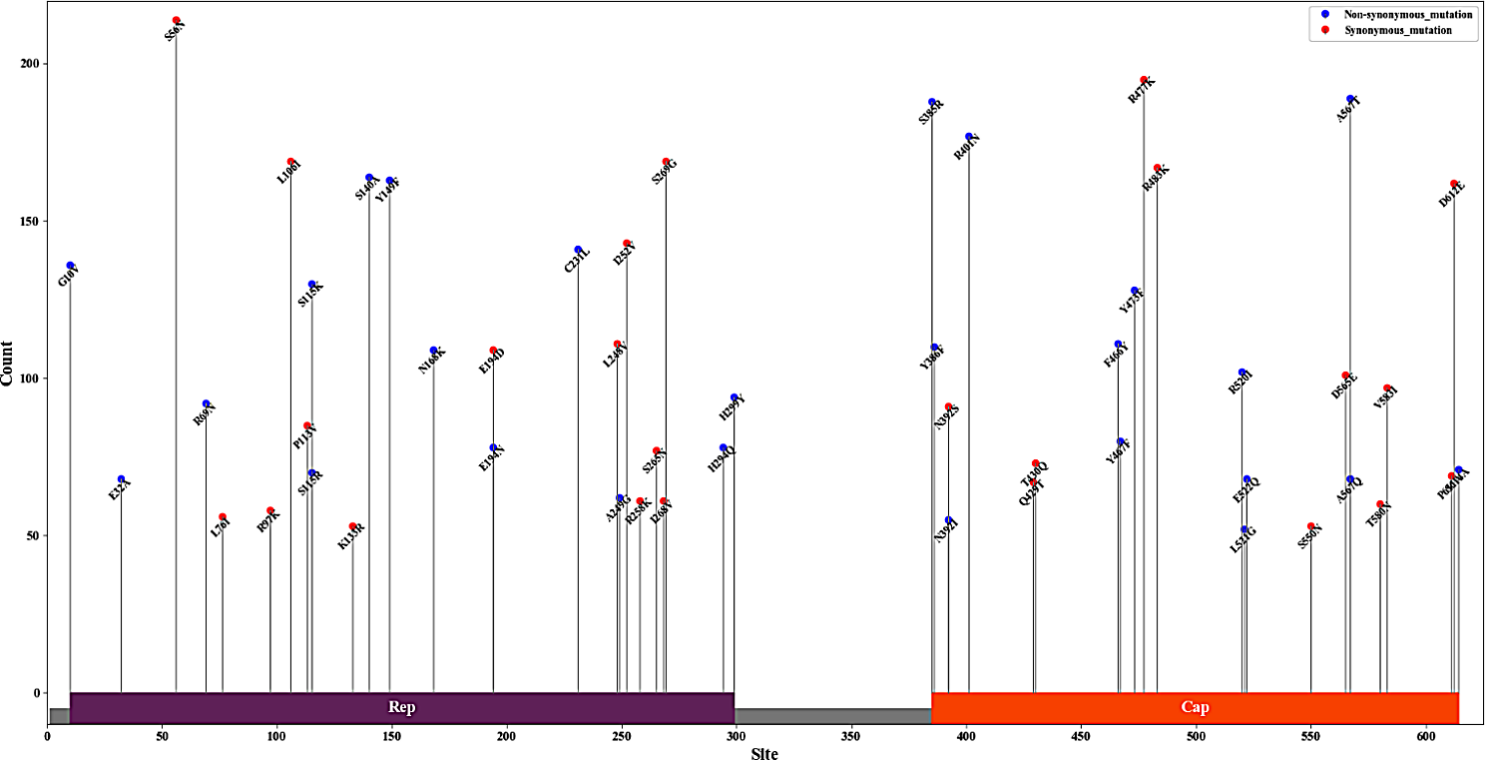
Amino acid alignment mutation landscape of CanineCV *Rep* and Cap proteins.

Amino acid alignment analysis revealed extensive substitutions at several positions, including V10G, A140S, L231C, and H299Y (Fig. 7). Specific genotypes also exhibited particular mutation sites: CanineCV-1 had Q5H, N68R, D124N, M163L, W203L, S256F, and R258T substitutions; CanineCV-4 had G10D; and CanineCV-6 had E34G and V37G. The Cap protein also exhibited multiple mutations, with different genotypes showing different sites: CanineCV-1 had L35M, F42K, K51E, P52Q, P56Q, F71S, K98I, L125Q, A134H, P151Q, G223S, M234VM, D252VM, and G267S substitutions; CanineCV-2 had R2C and A9T; CanineCV-3 had T238K; CanineCV-4 had Q27P, L89M, N156D, P202Q, Q215K, M231I, P236S, S244T, and V249I; CanineCV-5 had Q130H, K157T, Q230R, and I253S; and CanineCV-6 had Q2K, S14G, H38Q, and Y41H.

### Functional analysis of proteins from the adaptive evolutionary sites of CanineCV

Selection analysis using FEL, SLAC, FUBAR, and MEME identified specific sites on the *Rep* protein subject to positive selection in the CanineCV dataset. Significant p-values at these sites suggested positive selection and potential functional and adaptive changes in the CanineCV genome (Table 3). Sites 10, 16, 140, and 269 exhibited particularly low p-values across all analytical methods, providing robust evidence for positive selection. The p-values ≤ 0.001 across all methods for site 10 are highly indicative of positive selection and potential adaptive changes at this site. Similarly, site 269 showed consistently low p-values (0.006 to 0.000), emphasizing its importance in the adaptive evolution of CanineCV. Sites 16, 140, 149, and 164 also exhibited p-values that suggested potential roles in viral adaptation. These findings underscore the importance of these specific sites in shaping the evolutionary dynamics of CanineCV (Table A5 lists the positive amino acid mutation sites in the CanineCV *Rep* protein) and their potential influence on how it interacts with hosts and other factors that contribute to its adaptation.

**Table 3:** Probability (p-values of four selection analysis methods) of specific sites on the Rep protein of CanineCV being subject to positive selection

Amino acid site	FEL	SLAC	FUBAR	MEME
10	0.000	0.001	0.000	0.000
16	0.017	0.088	0.017	0.010
140	0.002	0.012	0.004	0.000
149	0.081	0.168	0.055	0.100
164	0.069	0.076	0.029	0.090
269	0.002	0.006	0.000	0.000

### Prediction of CanineCV protein modification sites

**Fig. 8:**
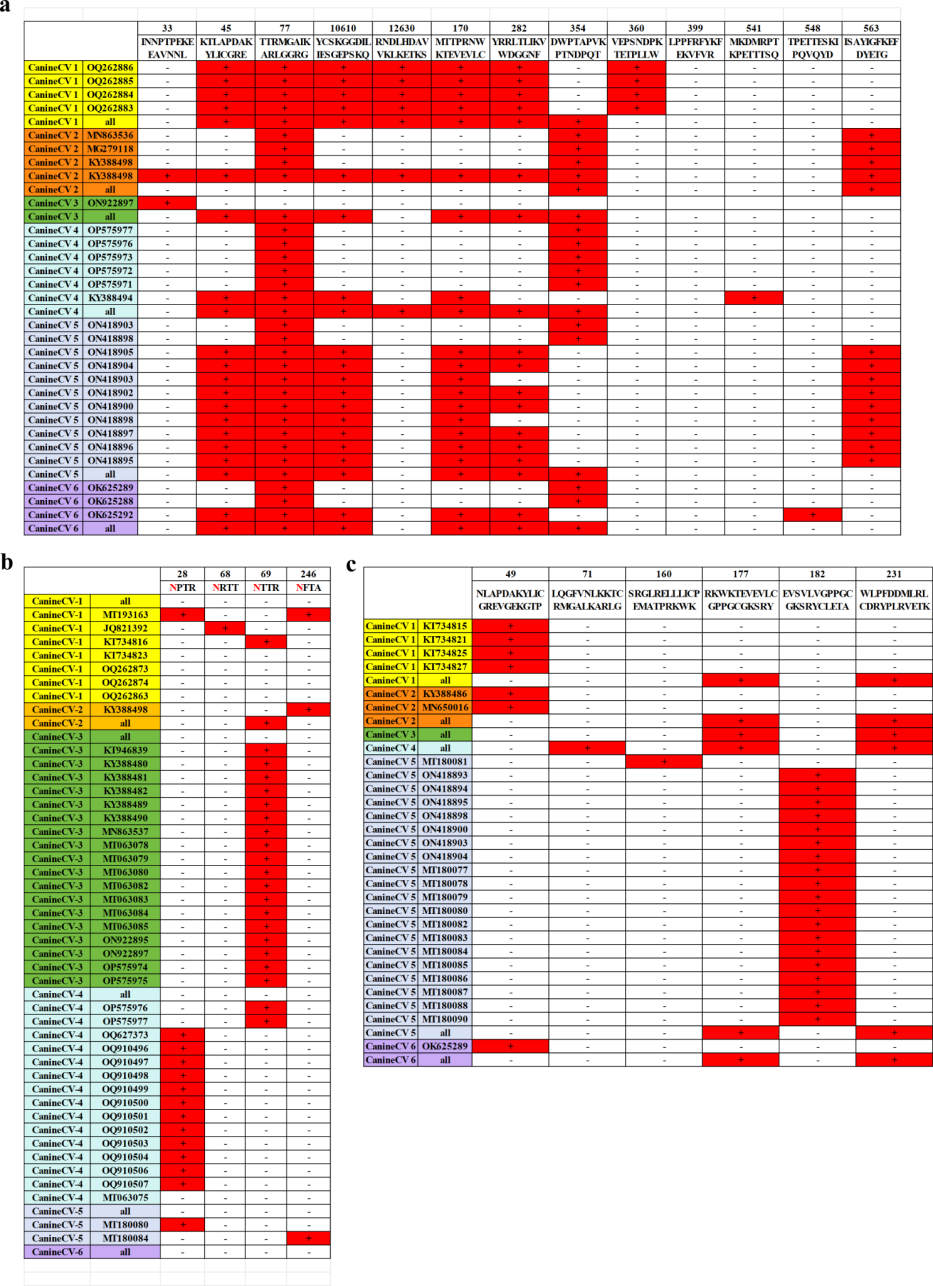
Predicted protein modification sites in the CanineCV genome: (**a**) SUMOylation sites, (**b**) Glycosylation sites, and (**c**) Palmitoylation sites. ‘+’: positive, ‘–’: negative

Our research predicted the protein modification site of the *Rep* gene of canine circovirus (Fig. 8a). CanineCV 1 strains show SUMOylation at positions 45, 77, 106–110, 126–130, 170, 282, 354 and 360, but not at positions 33, 399, 541, 548, and 563. CanineCV 2 strains show SUMOylation at position 354, but generally not at other positions. One strain of CanineCV 3 is SUMOylated only at position 33. CanineCV 4 demonstrates SUMOylation at positions 77, 106–110, and 170, but there is some variation among the strains. Apart from uniform modification at position 77, it can be concluded that the sequences at positions 77, 360, and 399 are consistently associated with the presence of CanineCV 5. All CanineCV 6 strains are SUMOylated at position 77, while varying at other positions.

Predicted sites of glycosylation are illustrated in Fig. 8b. Four CanineCV 1 strains show glycosylation at various single positions, while other strains show none. One strain of CanineCV 2 is glycosylated at position 246. CanineCV 3 strains are consistently glycosylated solely at position 69. Many strains of CanineCV 4 are glycosylated at position 28, but there are some exceptions to this modification. Two CanineCV 5 strains show glycosylation at positions 28 or 246. One CanineCV 6 strain strains lack glycosylation.

Predicted sites of palmitoylation are illustrated in Fig. 8c. CanineCV 1 strain KT734815-1, KT734821-1, KT734825-1, and KT734827-1 exhibit positive palmitoylation at position 49, while other sites are negative. CanineCV 2 strains KY388486-2 and MN650016-2 display positive palmitoylation at position 49, with other sites being negative. CanineCV 3 strains show positive palmitoylation at positions 177 and 231, with other sites being negative. CanineCV 4 strains exhibit positive palmitoylation at position 71, 177 and 231, with other sites being negative. CanineCV 5 strain MT180081-5 demonstrates positive palmitoylation at position 160, while other strains show a mixture of positive and negative palmitoylation sites. CanineCV 6 strain OK625289-6 displays positive palmitoylation at position 49, with other sites being negative.

These findings demonstrate significant variations in the sites of protein modification across different strains of CanineCV, suggesting potential differences in protein functions and interactions. Further research is required to elucidate the functional implications of these modifications in CanineCV biology.

### Mutational pressures shaping codon usage bias

**Fig. 9:**
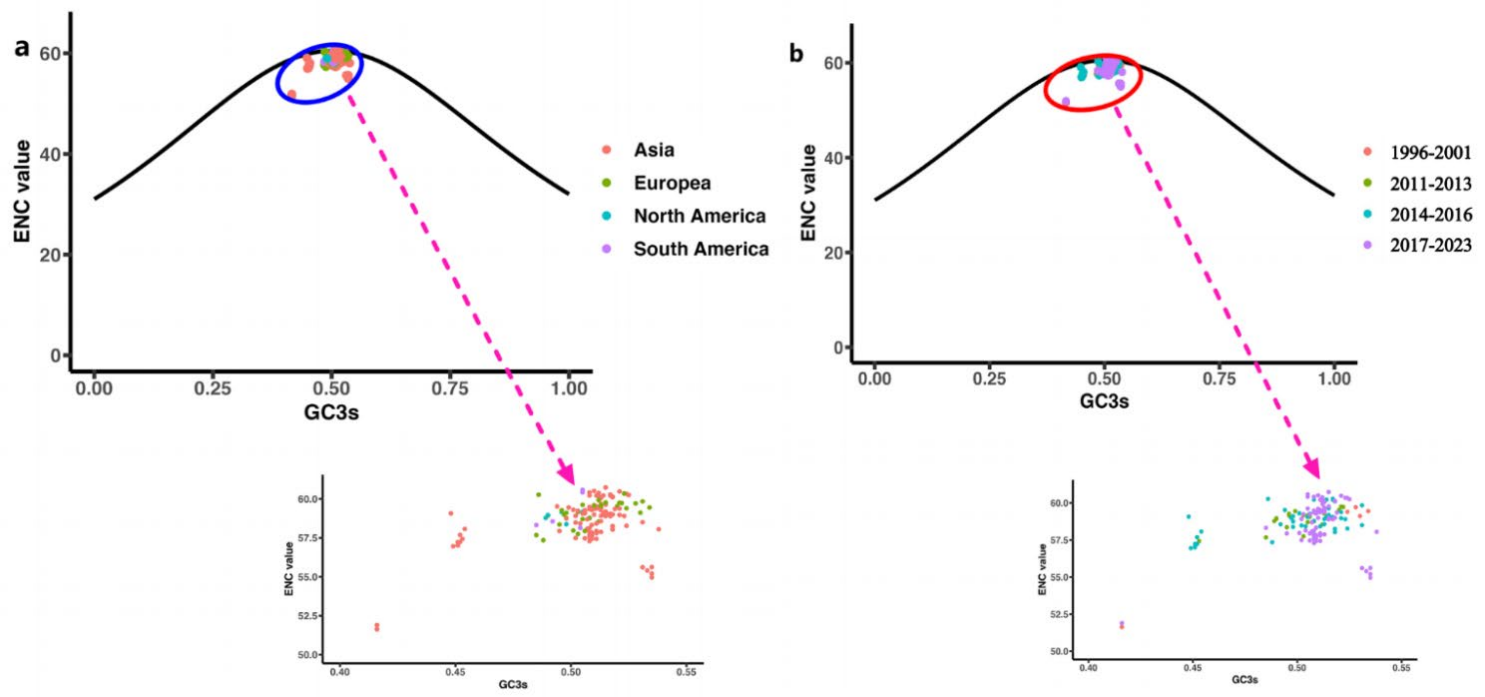
Effective number of codons (ENC) plots for the GC content in synonymous codons at the third position (GC3s) of CanineCV, color-coding samples by (**a**) geographical region and (**b**) year of isolation

GC3s ENC plots were generated based on geographical distribution (Fig. 9a) and year of isolation (Fig. 9b) of the sequences in order to elucidate the pattern of synonymous codon usage in CanineCV. All datapoints representing CanineCV strains in these plots were below the standard curve, indicating a codon usage bias in the virus. Notably, strains isolated from a given region, particularly Asia, did not cluster together, suggesting that mutational and other pressures impact the virus within a geographical region. Moreover, the ENC values of European strains were generally higher than those from Asia. The clustering of sequences from different regions and years (except for Asia in 2017) indicates that codon usage bias was influenced predominantly by factors beyond mutation pressure. The fact that many sequences did not align with the standard curve underscores the role of geographical distribution as an influencing factor and points to a complex and nuanced interplay of mutational pressures and other factors affecting synonymous codon usage bias in CanineCV.

## Discussion

Since its discovery in 2012 [15], CanineCV research has focused on genetic characterization of individual isolates alongside clinical and epidemiological investigations. This study provides updated information on recent CanineCV cases in China and reveals varying infection frequencies across geographic regions, ranging from 0 to 10%. This is consistent with, or in some instances higher than, the frequencies reported in previous studies in this country [22, 31]. Our study’s collection and prevalence data indicate that limited sample sizes in some provinces (such as Shanghai and Sichuan) may affect the reliability of prevalence estimates. This limitation highlights the need for more balanced and representative sampling across all regions to strengthen the study’s conclusions. Future studies should aim to collect more comprehensive data from underrepresented areas to provide a more accurate and generalizable understanding of CanineCV prevalence.

Six distinct groups within the CanineCV species were identified by phylogenetic analysis. CanineCV-1 is present in Europe [Italy [29], Germany [28]], North America [USA [16]], South America [Brazil [25], Colombia [24], Argentina [24]], and Asia [China [26, 27], Vietnam [50]]. CanineCV-2 is exclusively found in China and is yet to be reported elsewhere. This is considered a distinct genetic variant within China and ongoing research and surveillance are necessary to monitor potential changes in its distribution and to understand its implications for canine health. CanineCV-3 is found in China and Vietnam. CanineCV-4 is distributed mainly within China, although a strain has recently been discovered in Thailand [51]. CanineCV-5 has only been identified in Arctic foxes (*Vulpes lagopus*) and red foxes (*Vulpes vulpes*) in Norway and Great Britain [52], while CanineCV-6 is believed to be a new genotype identified in Iran [30].

The biological functioning of many viruses relies on N-linked glycosylation. Despite differences in hosts and modes of infection, the N-glycosylation sites of circoviruses are mostly conserved, suggesting they are associated with critical biological properties [53]. The present study supports this view as it predicts the presence of 16 conserved glycosylation sites in the genome of CanineCV. A study of the Sindbis virus demonstrated that the absence of either of its two E2 N-glycosylation sites led to increased replication and virulence in mammalian cells [54].

This study identified potential epitopes for positive selection in specific positions of the *Rep* and Cap proteins of CanineCV. These sites may play crucial roles in the virus’s ability to evade its host’s immune system, allowing it to continue to circulate and replicate. Specific sites within the Rep protein (crucial for viral replication and the regulation of the viral life cycle) exhibited strong signs of positive selection [55]. This suggests that CanineCV is evolving to enhance its replication efficiency, allowing the virus to replicate more effectively in diverse host environments, thereby increasing its fitness and survivability. Similarly, several sites in the Cap protein (responsible for forming the viral capsid that protects the viral genome and facilitates entry into host cells) showed evidence of positive selection. Positive selection in the Cap protein indicates ongoing adaptations that may improve the virus’s ability to infect different host species, enhance its ability to evade host immune responses, and increase its infectivity across various hosts [38].

Moreover, specific mutations in the *Rep* and Cap proteins were observed for each genotype, which warrants further investigation to elucidate the role of ancestral sequence variation [16] in the global evolution of CanineCV (for example, addressing KC241983 as a phylogenetic outgroup). Currently, MG266899/China/CD17/2016 is the only CanineCV-1 strain being detected in Asia, while MG737385/Thailand/CP191st/2016 is the only CanineCV-3 strain circulating outside China, having been detected in Thailand [56]. Various countries, including Italy, Argentina, Brazil, China, South Korea, and Thailand, have experienced co-circulation and maintenance of multiple CanineCV transmission chains over several years, while also importing novel chains from other countries.

This study gives an insight into the origin, evolution, and global trajectory of CanineCV and sheds light on its expanding range of hosts and geographic reach. Its findings contribute to a nuanced understanding of CanineCV epidemiology and offer valuable data on the current global distribution of the virus. This research has significant implications for the management of CanineCV through enhanced diagnostics, vaccine development, targeted surveillance, and public health strategies. An understanding of the evolutionary dynamics of CanineCV can aid development of diagnostic tools that detect a broader range of viral strains, thus enhancing early detection. Insight into the virus’s population changes can guide the development of vaccines that target the prevailing strains, while surveillance programs focusing on high-risk periods and regions can be designed. Public health measures can also be optimized to prevent and manage outbreaks in ways that enhance disease management in canines.

This study provides insight into the evolutionary dynamics and expanding host range of CanineCV. Several key evolutionary pressures are hypothesized to drive the diversification and adaptation of CanineCV across hosts and regions. For example, host immune responses exert selective pressure on the virus, driving it to evolve mechanisms to evade or suppress those responses, as evidenced by positive selection in the Rep and Cap proteins. Environmental variables (e.g., temperature, humidity, and presence of other pathogens) can create niches that select for viral variants with specific adaptive traits. Host switching and co-evolution can also drive rapid genetic changes and diversification as the virus adapts to new host species. Genetic recombination, identified in this study, introduces beneficial mutations and novel gene combinations that facilitate adaptation to various hosts and ecological niches. Changes in host population size and structure, including population bottlenecks, can also influence the genetic diversity of CanineCV. Bottlenecks may reduce genetic diversity, while subsequent expansions can lead to the rapid spread of advantageous mutations. Consideration of these evolutionary pressures yields greater understanding of the factors driving the diversification and adaptation of CanineCV, which is crucial for developing effective strategies for monitoring, controlling, and preventing the spread of the virus across host species and regions.

## Conclusions

This study provides valuable insight into the evolutionary dynamics and expanding host range of CanineCV. To further elucidate the genetic evolution, pathogenicity, and epidemiology of CanineCV, several future research directions are suggested: (i) long-term surveillance and longitudinal studies across various regions and host species to monitor evolutionary changes over time, (ii) experimental studies of the functional roles of positively selected sites in the Rep and Cap proteins to understand their influence on viral replication, host immune evasion, and pathogenicity, (iii) exploration of the molecular mechanisms of host switching and co-evolution to determine how the virus adapts to new hosts and which evolutionary pressures are involved, (iv) analysis of recombination events to elucidate their impact on genetic diversity and viral evolution, (v) comprehensive epidemiological studies to map the geographic distribution and prevalence of CanineCV and identify high-risk areas and the factors contributing to virus spread, and (vi) development of sensitive diagnostic tools and effective vaccines, focusing on creation of broad-spectrum vaccines that provide immunity against diverse strains. Addressing these research gaps could enhance our understanding of CanineCV’s genetic evolution, pathogenicity, and epidemiology and lead to better strategies for monitoring, controlling, and preventing the spread of this virus.

### Electronic supplementary material

Below is the link to the electronic supplementary material.

Supplementary Material 1

## Data Availability

No datasets were generated or analysed during the current study.
