# Supplementary material for: Epidemiological and evolutionary analysis of canine circovirus from 1996 to 2023
Source: BMC Vet Res. 2024 Jul 20;20:328. doi: 10.1186/s12917-024-04186-6 (PMC11264901; doi:10.1186/s12917-024-04186-6)
Supplement: Supplementary file 1 — Supplementary Material 1 [file 12917_2024_4186_MOESM1_ESM.docx]

**Epidemiological and evolutionary analysis of canine circovirus from 1996 to 2023**

**Additional Files**

**Table A1** Genomes sequenced from Canine circovirus-positive dogs (n = 64).

| **GenBank accession no.** | **ID** | **Group** | **Sample type** | **Year** | **Province** |
| --- | --- | --- | --- | --- | --- |
| MG279121 | 186 | CanineCV 2 | serum | 2017 | Guangxi |
| MG279133 | 394 | CanineCV 2 | serum | 2018 | Guangxi |
| MG279140 | 205 | CanineCV 2 | serum | 2018 | Guangxi |
| MN689726 | CQ76 | CanineCV 2 | serum | 2018 | Guangxi |
| MN709512 | CQ79 | CanineCV 2 | serum | 2018 | Guangxi |
| MN709511 | CQ82 | CanineCV 2 | serum | 2018 | Guangxi |
| MG279118 | 102 | CanineCV 2 | serum | 2017 | Guangxi |
| MG279119 | 199 | CanineCV 2 | serum | 2017 | Chongqing |
| MG279120 | 198 | CanineCV 2 | serum | 2017 | Guangxi |
| MG279122 | 176 | CanineCV 2 | serum | 2017 | Guangxi |
| MG279123 | 185 | CanineCV 2 | serum | 2017 | Guangxi |
| MG279124 | 183 | CanineCV 2 | serum | 2017 | Guangxi |
| MG279125 | 182 | CanineCV 2 | serum | 2017 | Guangxi |
| MG279126 | 181 | CanineCV 2 | serum | 2017 | Guangxi |
| MG279127 | 180 | CanineCV 2 | serum | 2017 | Guangxi |
| MG279128 | 179 | CanineCV 2 | serum | 2017 | Guangxi |
| MG279129 | 178 | CanineCV 2 | serum | 2017 | Guangxi |
| MG279130 | 177 | CanineCV 2 | serum | 2017 | Guangxi |
| MG279131 | 202 | CanineCV 2 | serum | 2017 | Chongqing |
| MG279132 | 398 | CanineCV 2 | serum | 2018 | Guangxi |
| MG279134 | 394 | CanineCV 2 | serum | 2018 | Guangxi |
| MG279135 | 201 | CanineCV 2 | serum | 2017 | Chongqing |
| MG279136 | 391 | CanineCV 2 | serum | 2018 | Guangxi |
| MG279137 | 390 | CanineCV 2 | serum | 2018 | Guangxi |
| MG279138 | 388 | CanineCV 2 | serum | 2018 | Guangxi |
| MG279139 | 384 | CanineCV 2 | serum | 2018 | Guangxi |
| MG279141 | 204 | CanineCV 2 | serum | 2018 | Guangxi |
| MN650013 | LA_3/2018 | CanineCV 2 | serum | 2018 | Guangxi |
| MN650014 | LA_5/2018 | CanineCV 2 | serum | 2019 | Neimenggu |
| MN650015 | LA_6/2018 | CanineCV 2 | serum | 2019 | Neimenggu |
| MN650016 | LA_13/2018 | CanineCV 2 | serum | 2019 | Guangxi |
| MN650017 | LA_21/2018 | CanineCV 2 | serum | 2018 | Guangxi |
| MN650018 | LA_23/2018 | CanineCV 2 | serum | 2018 | Guangxi |
| MN650019 | LA_H20/2018 | CanineCV 2 | serum | 2018 | Guangxi |
| MN650020 | LA_H21/2018 | CanineCV 2 | serum | 2018 | Guangxi |
| MN650021 | LA_H28/2018 | CanineCV 2 | serum | 2019 | Guangxi |
| MN650022 | LA_H29/2018 | CanineCV 2 | serum | 2018 | Guangxi |
| MN650025 | GP_P4/2019 | CanineCV 2 | serum | 2019 | Guangxi |
| MN650026 | BS_Q4/2018 | CanineCV 2 | serum | 2019 | Jiangsu |
| MN650027 | BS_Q6/2018 | CanineCV 2 | serum | 2019 | Jiangsu |
| MN650028 | BS_Q14/2018 | CanineCV 2 | serum | 2019 | Jilin |
| MN650029 | BS_Q32/2018 | CanineCV 2 | serum | 2019 | Jilin |
| MN650030 | BS_Q38/2018 | CanineCV 2 | serum | 2019 | Shandong |
| MN650031 | BS_Q66/2018 | CanineCV 2 | serum | 2019 | Shandong |
| MT063068 | LA_1/2018 | CanineCV 2 | serum | 2018 | Guangxi |
| MT063069 | LA_16/2018 | CanineCV 2 | serum | 2018 | Guangxi |
| MT063070 | LA_H22/2018 | CanineCV 2 | serum | 2018 | Guangxi |
| MT063071 | LA_H19/2018 | CanineCV 2 | serum | 2018 | Guangxi |
| MT063076 | JL21 | CanineCV 2 | serum | 2019 | Beijing |
| MT063086 | BS_Q7/2018 | CanineCV 2 | serum | 2019 | Jiangsu |
| MT063087 | BS_Q10/2018 | CanineCV 2 | serum | 2019 | Jilin |
| MT063088 | BS_Q44/2019 | CanineCV 2 | serum | 2019 | Shandong |
| MT063078 | JL9 | CanineCV 3 | serum | 2017 | Guangxi |
| MT063079 | SD11 | CanineCV 3 | serum | 2017 | Guangxi |
| MT063080 | SD16 | CanineCV 3 | serum | 2017 | Guangxi |
| MT063082 | NN20/2019 | CanineCV 3 | serum | 2017 | Guangxi |
| MT063083 | K3/2019 | CanineCV 3 | serum | 2017 | Guangxi |
| MT063084 | K8/2019 | CanineCV 3 | serum | 2017 | Guangxi |
| MT063085 | K31/2019 | CanineCV 3 | serum | 2017 | Guangxi |
| MN650023 | NM_N73/2019 | CanineCV 4 | serum | 2017 | Guangxi |
| MN650024 | NM_N91/2019 | CanineCV 4 | serum | 2017 | Guangxi |
| MT063075 | BJ-1-2019 | CanineCV 4 | serum | 2017 | Guangxi |
| MT063077 | JL19 | CanineCV 4 | serum | 2017 | Guangxi |
| MT063081 | SD8 | CanineCV 4 | serum | 2017 | Guangxi |

**Table A2. Details of gene sequences of circovirus strain.**

| **Accession** | **Length** | **Organism** | **Strain** | **Collection region** | **Collection date** |
| --- | --- | --- | --- | --- | --- |
| GU325756 | 1767 | Porcine circovirus 2 | Unknown | China | 2009 |
| NC_055122 | 1761 | Circovirus siksparnis | Unknown | China | 2011 |
| NC_031753 | 2000 | Porcine circovirus 3 | 29160 | USA: North Carolina | 2015 |
| MH603554 | 2000 | Porcine circovirus 3 | KSU-KS-2017-PCV3-53 | USA | 2017 |
| OP963683 | 1816 | Bat circovirus BtSY1 | Bat/2018/S18CXBatR22 | China: Yunnan | 2018 |
| NC_021206 | 1798 | Bat associated circovirus 2 | Unknown | Myanmar | 2008/11/1 |
| NC_038385 | 1862 | Bat associated circovirus 1 | Unknown | Myanmar | 2008/11/1 |
| NC_039033 | 1900 | Bat associated circovirus 9 | Unknown | China | 2010/12/1 |
| NC_038390 | 2069 | Bat associated circovirus 8 | Unknown | China | 2012/12/1 |
| MH760365 | 1798 | Bat circovirus | BtRf-CV/YN256/2013 | China | 2013/5/3 |
| NC_028045 | 1767 | Tadarida brasiliensis circovirus 1 | Unknown | Brazil | 2013/6/12 |
| NC_023885 | 1753 | Mink circovirus | MiCV-DL13 | China | 2013/10/30 |
| MK070856 | 1764 | Bat associated circovirus | Unknown | China | 2014/7/24 |
| MH760364 | 1761 | Bat circovirus | YNGL167/2015 | China | 2015/12/12 |
| NC_077109 | 1967 | Wolvfec circovius | Unknown | USA | 2018/3/17 |
| NC_055580 | 1770 | Porcine circovirus 4 | HNU-AHG1-2019 | China | 2019/2/1 |
| KX756986 | 2113 | Bat circovirus | Unknown | China | Unknown |

**Table A3. Details of gene sequences of Canine circovirus strains.**

| **Accession** | **Isolate** | **Length** | **Location** | **Host** | **Collection Date** |
| --- | --- | --- | --- | --- | --- |
| MT180083 | 02/1996 | 2063 | Norway | Vulpes lagopus | 1996 |
| MT180084 | 47/1997 | 2063 | Norway | Vulpes lagopus | 1997 |
| MT180087 | 57/1997 | 2063 | Norway | Vulpes lagopus | 1997 |
| MT180089 | 70/1997 | 2063 | Norway | Vulpes lagopus | 1997 |
| MT180090 | 79/1997 | 2063 | Norway | Vulpes lagopus | 1997 |
| MT180086 | 55/1998 | 2063 | Norway | Vulpes lagopus | 1998 |
| MT180085 | 52/1999 | 2063 | Norway | Vulpes lagopus | 1999 |
| MT180088 | 65/1999 | 2063 | Norway | Vulpes lagopus | 1999 |
| MT193159 | CanineCV-800/2009 | 2063 | Italy | Canis lupus familiaris | 2009 |
| MZ407653 | Fox61/Italy/VulpesVulpes/2009 | 2063 | Italy | Vulpes vulpes | 2009 |
| MT193160 | CanineCV-115/2010 | 2063 | Italy | Canis lupus familiaris | 2010 |
| MT193161 | CanineCV-121/2010 | 2063 | Italy | Canis lupus familiaris | 2010 |
| MT193162 | CanineCV-540/2010 | 2063 | Italy | Canis lupus familiaris | 2010 |
| JQ821392 | 214 | 2063 | USA | Canis lupus familiaris | 2011 |
| MK424788 | CanineCV D1056 | 2063 | Brazil | Canis lupus familiaris | 2013 |
| MT193163 | CanineCV-570/2013 | 2063 | Italy | Canis lupus familiaris | 2013 |
| KT946839 | JZ98/2014 | 2063 | China | Canis lupus | 2014 |
| KY388481 | JZ50 | 2063 | China | Canis lupus familiaris | 2014 |
| KY388490 | JZ82 | 2063 | China | Canis lupus familiaris | 2014 |
| KY388489 | JZ85 | 2063 | China | Canis lupus familiaris | 2014 |
| KY388488 | LA128 | 2063 | China | Canis lupus familiaris | 2014 |
| KY388487 | LA237 | 2064 | China | Canis lupus familiaris | 2014 |
| KY388486 | LA280 | 2063 | China | Canis lupus familiaris | 2014 |
| KP941114 | 55590 | 2055 | Croatia | Vulpes vulpes | 2014 |
| MT193164 | CanineCV-608/2014 | 2063 | Italy | Canis lupus familiaris | 2014 |
| MT180078 | 19/2014 | 2063 | Norway | Vulpes vulpes | 2014 |
| MT180079 | 27/2014 | 2063 | Norway | Vulpes vulpes | 2014 |
| KY388480 | GL33 | 2063 | China | Canis lupus familiaris | 2015 |
| KY388482 | GL51 | 2063 | China | Canis lupus familiaris | 2015 |
| KY388485 | WM46 | 2064 | China | Canis lupus familiaris | 2015 |
| KY388484 | WM48 | 2064 | China | Canis lupus familiaris | 2015 |
| KY388483 | WM60 | 2064 | China | Canis lupus familiaris | 2015 |
| KY388493 | WM62 | 2064 | China | Canis lupus familiaris | 2015 |
| KY388491 | WM63 | 2064 | China | Canis lupus familiaris | 2015 |
| KY388492 | WM66 | 2064 | China | Canis lupus familiaris | 2015 |
| KY388503 | WM72 | 2064 | China | Canis lupus familiaris | 2015 |
| KY388502 | WM74 | 2064 | China | Canis lupus familiaris | 2015 |
| KY388501 | WM76 | 2064 | China | Canis lupus familiaris | 2015 |
| KY388500 | WM77 | 2064 | China | Canis lupus familiaris | 2015 |
| KY388499 | WM79 | 2064 | China | Canis lupus familiaris | 2015 |
| KY388498 | WM83 | 2063 | China | Canis lupus familiaris | 2015 |
| KY388497 | WM84 | 2064 | China | Canis lupus familiaris | 2015 |
| KY388496 | XXT242 | 2063 | China | Canis lupus familiaris | 2015 |
| KY388495 | XXT243 | 2063 | China | Canis lupus familiaris | 2015 |
| MT180077 | 01/2015 | 2063 | Norway | Vulpes vulpes | 2015 |
| KY388494 | YL11 | 2063 | China | Canis lupus familiaris | 2016 |
| MT193165 | CanineCV-1203/2016 | 2063 | Italy | Canis lupus familiaris | 2016 |
| MT193166 | CanineCV-1367/2016 | 2063 | Italy | Canis lupus familiaris | 2016 |
| MN689726 | CQ76 | 2062 | China | Canis lupus familiaris | 2017 |
| MN709512 | CQ79 | 2062 | China | Canis lupus familiaris | 2017 |
| MN709511 | CQ82 | 2062 | China | Canis lupus familiaris | 2017 |
| MG279118 | 102 | 2063 | China | Unknown | 2017 |
| MG279122 | 176 | 2063 | China | Unknown | 2017 |
| MG279130 | 177 | 2063 | China | Unknown | 2017 |
| MG279129 | 178 | 2063 | China | Unknown | 2017 |
| MG279128 | 179 | 2063 | China | Unknown | 2017 |
| MG279127 | 180 | 2063 | China | Unknown | 2017 |
| MG279126 | 181 | 2063 | China | Unknown | 2017 |
| MG279125 | 182 | 2063 | China | Unknown | 2017 |
| MG279124 | 183 | 2063 | China | Unknown | 2017 |
| MG279123 | 185 | 2063 | China | Unknown | 2017 |
| MG279121 | 186 | 2063 | China | Unknown | 2017 |
| MG279120 | 198 | 2063 | China | Unknown | 2017 |
| MG279119 | 199 | 2063 | China | Unknown | 2017 |
| MG279131 | 202 | 2063 | China | Unknown | 2017 |
| MG279141 | 204 | 2063 | China | Unknown | 2017 |
| MG279140 | 205 | 2063 | China | Unknown | 2017 |
| MG279139 | 384 | 2064 | China | Unknown | 2017 |
| MG279138 | 388 | 2064 | China | Unknown | 2017 |
| MG279137 | 390 | 2064 | China | Unknown | 2017 |
| MG279136 | 391 | 2064 | China | Unknown | 2017 |
| MG279134 | 394 | 2064 | China | Unknown | 2017 |
| MG279133 | 395 | 2064 | China | Unknown | 2017 |
| MG279132 | 398 | 2064 | China | Unknown | 2017 |
| MW829201 | CanineCV-447/2017 | 2063 | Italy | Canis lupus | 2017 |
| MW829202 | CanineCV-448/2017 | 2063 | Italy | Canis lupus | 2017 |
| MW829203 | CanineCV-449/2017 | 2063 | Italy | Canis lupus | 2017 |
| MW829204 | CanineCV-450/2017 | 2063 | Italy | Canis lupus | 2017 |
| MT180080 | 64/2017 | 2063 | Norway | Vulpes vulpes | 2017 |
| MT180081 | 73/2017 | 2063 | Norway | Vulpes vulpes | 2017 |
| MT180082 | 77/2017 | 2063 | Norway | Vulpes vulpes | 2017 |
| MT740194 | VN-1 | 2063 | Viet Nam | Canis lupus familiaris | 2017 |
| MT740195 | VN-2 | 2063 | Viet Nam | Canis lupus familiaris | 2017 |
| MT740198 | VN-3 | 2063 | Viet Nam | Canis lupus familiaris | 2017 |
| MT740201 | VN-4 | 2063 | Viet Nam | Canis lupus familiaris | 2017 |
| MT740199 | VN-5 | 2063 | Viet Nam | Canis lupus familiaris | 2017 |
| ON418903 | FGB10 | 2063 | Canada | Vulpes vulpes | 2018 |
| ON418905 | FGB6 | 2063 | Canada | Vulpes vulpes | 2018 |
| ON418904 | FGB7 | 2063 | Canada | Vulpes vulpes | 2018 |
| ON418901 | FLC21 | 2063 | Canada | Vulpes vulpes | 2018 |
| ON418900 | FLC27 | 2063 | Canada | Vulpes vulpes | 2018 |
| ON418899 | FLC30 | 2063 | Canada | Vulpes vulpes | 2018 |
| ON418902 | FLC8 | 2063 | Canada | Vulpes vulpes | 2018 |
| ON418898 | FNA4 | 2063 | Canada | Vulpes vulpes | 2018 |
| ON418897 | FNA7 | 2063 | Canada | Vulpes vulpes | 2018 |
| ON418896 | FNA8 | 2063 | Canada | Vulpes vulpes | 2018 |
| ON418895 | FX7 | 2063 | Canada | Vulpes vulpes | 2018 |
| MT063087 | BS_Q10/2018 | 2064 | China | Canis lupus familiaris | 2018 |
| MN650028 | BS_Q14/2018 | 2064 | China | Canis lupus familiaris | 2018 |
| MN650029 | BS_Q32/2018 | 2064 | China | Canis lupus familiaris | 2018 |
| MN650030 | BS_Q38/2018 | 2064 | China | Canis lupus familiaris | 2018 |
| MN650026 | BS_Q4/2018 | 2064 | China | Canis lupus familiaris | 2018 |
| MN650027 | BS_Q6/2018 | 2064 | China | Canis lupus familiaris | 2018 |
| MN650031 | BS_Q66/2018 | 2064 | China | Canis lupus familiaris | 2018 |
| MT063086 | BS_Q7/2018 | 2064 | China | Canis lupus familiaris | 2018 |
| MT063078 | JL9 | 2063 | China | Canis lupus familiaris | 2018 |
| MT063068 | LA_1/2018 | 2064 | China | Canis lupus familiaris | 2018 |
| MN650016 | LA_13/2018 | 2064 | China | Canis lupus familiaris | 2018 |
| MT063069 | LA_16/2018 | 2064 | China | Canis lupus familiaris | 2018 |
| MN650017 | LA_21/2018 | 2064 | China | Canis lupus familiaris | 2018 |
| MN650018 | LA_23/2018 | 2064 | China | Canis lupus familiaris | 2018 |
| MN650013 | LA_3/2018 | 2064 | China | Canis lupus familiaris | 2018 |
| MN650014 | LA_5/2018 | 2064 | China | Canis lupus familiaris | 2018 |
| MN650015 | LA_6/2018 | 2064 | China | Canis lupus familiaris | 2018 |
| MT063071 | LA_H19/2018 | 2064 | China | Canis lupus familiaris | 2018 |
| MN650019 | LA_H20/2018 | 2064 | China | Canis lupus familiaris | 2018 |
| MN650020 | LA_H21/2018 | 2064 | China | Canis lupus familiaris | 2018 |
| MT063070 | LA_H22/2018 | 2064 | China | Canis lupus familiaris | 2018 |
| MN650021 | LA_H28/2018 | 2064 | China | Canis lupus familiaris | 2018 |
| MN650022 | LA_H29/2018 | 2064 | China | Canis lupus familiaris | 2018 |
| MT293519 | MED-1 | 2063 | Colombia | Canis lupus familiaris | 2018 |
| MT293520 | MED-2 | 2063 | Colombia | Canis lupus familiaris | 2018 |
| MT293521 | MED-3 | 2063 | Colombia | Canis lupus familiaris | 2018 |
| MW829205 | CanineCV-454/2018 | 2063 | Italy | Canis lupus | 2018 |
| MW829206 | CanineCV-457/2018 | 2063 | Italy | Canis lupus | 2018 |
| MW829207 | CanineCV-458/2018 | 2063 | Italy | Canis lupus | 2018 |
| MT740196 | VN-6 | 2063 | Viet Nam | Canis lupus familiaris | 2018 |
| MT740197 | VN-7 | 2063 | Viet Nam | Canis lupus familiaris | 2018 |
| MT740200 | VN-8 | 2063 | Viet Nam | Canis lupus familiaris | 2018 |
| MT063075 | BJ-1-2019 | 2063 | China | Canis lupus familiaris | 2019 |
| MT063074 | BJ-3-2019 | 2063 | China | Canis lupus familiaris | 2019 |
| MT063073 | BJ-4-2019 | 2063 | China | Canis lupus familiaris | 2019 |
| MT063072 | BJ-6-2019 | 2063 | China | Canis lupus familiaris | 2019 |
| MT063088 | BS_Q44/2019 | 2064 | China | Canis lupus familiaris | 2019 |
| MN650025 | GP_P4/2019 | 2064 | China | Canis lupus familiaris | 2019 |
| MT063077 | JL19 | 2063 | China | Canis lupus familiaris | 2019 |
| MT063076 | JL21 | 2063 | China | Canis lupus familiaris | 2019 |
| MT063083 | K3/2019 | 2063 | China | Canis lupus familiaris | 2019 |
| MT063085 | K31/2019 | 2063 | China | Canis lupus familiaris | 2019 |
| MT063084 | K8/2019 | 2063 | China | Canis lupus familiaris | 2019 |
| MN650023 | NM_N73/2019 | 2063 | China | Canis lupus familiaris | 2019 |
| MN650024 | NM_N91/2019 | 2063 | China | Canis lupus familiaris | 2019 |
| MT063082 | NN20/2019 | 2063 | China | Canis lupus familiaris | 2019 |
| MT063079 | SD11 | 2063 | China | Canis lupus familiaris | 2019 |
| MT063080 | SD16 | 2063 | China | Canis lupus familiaris | 2019 |
| MT063081 | SD8 | 2063 | China | Canis lupus familiaris | 2019 |
| OM136006 | CanineCV4 | 2063 | China | Canis lupus familiaris | 2019 |
| ON922901 | 266 | 2063 | China | canine | 2020 |
| OQ198055 | CircoviridaeDogfe426C1 | 2063 | China | Canis lupus familiaris | 2021 |
| OQ198052 | CircoviridaeDogfe444C1 | 2054 | China | Canis lupus familiaris | 2021 |
| OP575971 | SH-1/2022 | 2063 | China | Canis lupus familiaris | 2021 |
| OP575972 | SH-2/2022 | 2063 | China | Canis lupus familiaris | 2021 |
| OP575973 | SH-3/2022 | 2063 | China | Canis lupus familiaris | 2021 |
| OQ377115 | MED_23 | 2062 | Colombia | Canis lupus familiaris | 2021 |
| OQ377116 | MED_24 | 2062 | Colombia | Canis lupus familiaris | 2021 |
| OQ377117 | MED_25 | 2062 | Colombia | Canis lupus familiaris | 2021 |
| OQ262862 | 12 | 2063 | Namibia | Canis lupus familiaris | 2021 |
| OQ262868 | 182 | 2063 | Namibia | Canis lupus familiaris | 2021 |
| OQ262882 | J31 | 2063 | Namibia | Black Backed Jackal | 2021 |
| OQ262883 | J38 | 2063 | Namibia | Black Backed Jackal | 2021 |
| OQ262884 | J39 | 2063 | Namibia | Black Backed Jackal | 2021 |
| OQ262885 | J40 | 2063 | Namibia | Black Backed Jackal | 2021 |
| OQ262886 | J45 | 2063 | Namibia | Black Backed Jackal | 2021 |
| OQ262878 | 415 | 2063 | Namibia | Canis lupus familiaris | 2021 |
| OQ262879 | 426 | 2063 | Namibia | Canis lupus familiaris | 2021 |
| OQ262889 | 715 | 2063 | Namibia | Canis lupus familiaris | 2021 |
| OQ262888 | 716 | 2063 | Namibia | Canis lupus familiaris | 2021 |
| OQ262887 | 723 | 2063 | Namibia | Canis lupus familiaris | 2021 |
| OQ262891 | 734 | 2063 | Namibia | Canis lupus familiaris | 2021 |
| OQ262877 | 358 | 2063 | Namibia | Canis lupus familiaris | 2021 |
| OP575983 | FS-1/2022 | 2063 | China | Canis lupus familiaris | 2022 |
| OP575984 | FS-2/2022 | 2063 | China | Canis lupus familiaris | 2022 |
| OP575985 | FS-3/2022 | 2063 | China | Canis lupus familiaris | 2022 |
| OP575974 | GZ-1/2022 | 2063 | China | Canis lupus familiaris | 2022 |
| OP575975 | GZ-2/2022 | 2063 | China | Canis lupus familiaris | 2022 |
| OP575976 | GZ-3/2022 | 2063 | China | Canis lupus familiaris | 2022 |
| OP575977 | GZ-4/2022 | 2063 | China | Canis lupus familiaris | 2022 |
| OP575978 | GZ-5/2022 | 2063 | China | Canis lupus familiaris | 2022 |
| OP575979 | GZ-6/2022 | 2063 | China | Canis lupus familiaris | 2022 |
| OP575980 | GZ-7/2022 | 2063 | China | Canis lupus familiaris | 2022 |
| OP575981 | GZ-8/2022 | 2063 | China | Canis lupus familiaris | 2022 |
| OP575982 | GZ-9/2022 | 2063 | China | Felis catus | 2022 |
| OQ262863 | 133 | 2063 | Namibia | Canis lupus familiaris | 2022 |
| OQ262864 | 161 | 2063 | Namibia | Canis lupus familiaris | 2022 |
| OQ262865 | 170 | 2063 | Namibia | Canis lupus familiaris | 2022 |
| OQ262866 | 175 | 2063 | Namibia | Canis lupus familiaris | 2022 |
| OQ262867 | 180 | 2063 | Namibia | Canis lupus familiaris | 2022 |
| OQ262869 | 196 | 2063 | Namibia | Canis lupus familiaris | 2022 |
| OQ262880 | 590 | 2063 | Namibia | Canis lupus familiaris | 2022 |
| OQ262881 | 595 | 2063 | Namibia | Canis lupus familiaris | 2022 |
| OQ262890 | 686 | 2063 | Namibia | Canis lupus familiaris | 2022 |
| OQ262870 | 206 | 2063 | Namibia | Canis lupus familiaris | 2022 |
| OQ262871 | 207 | 2063 | Namibia | Canis lupus familiaris | 2022 |
| OQ262872 | 216 | 2063 | Namibia | Canis lupus familiaris | 2022 |
| OQ262873 | 256 | 2063 | Namibia | Canis lupus familiaris | 2022 |
| OQ262874 | 257 | 2063 | Namibia | Canis lupus familiaris | 2022 |
| OQ262875 | 270 | 2063 | Namibia | Canis lupus familiaris | 2022 |
| OQ262876 | 284 | 2063 | Namibia | Canis lupus familiaris | 2022 |
| ON418893 | NP28 | 2063 | Canada | Canis lupus familiaris | 2022 |
| ON418894 | NP24 | 2063 | Canada | Canis lupus familiaris | 2022 |
| ON922897 | 313 | 2063 | China | Canis lupus | 2022 |
| ON922895 | 356 | 2063 | China | Canis lupus familiaris | 2022 |
| ON922896 | 109 | 2063 | China | Canis lupus | 2011/9/1 |
| MN863537 | JS-1/2019 | 2063 | China | Meles meles | 2011/10/1 |
| KC241982 | UCD1-1698 | 2063 | USA | Canis lupus | 2011/10/1 |
| KC241984 | UCD2-32162 | 2063 | USA | Canis lupus | 2011/10/1 |
| KC241983 | UCD3-478 | 2063 | USA | Canis lupus | 2011/10/1 |
| KT734814 | TE4016-13 | 2063 | Italy | Canis lupus | 2013/2/1 |
| KT734820 | AZ663/1-13 | 2063 | Italy | Canis lupus familiaris | 2013/2/1 |
| KT734824 | AZ663/2-13 | 2063 | Italy | Canis lupus familiaris | 2013/2/1 |
| KT734821 | TE6685/1-13 | 2063 | Italy | Canis lupus familiaris | 2013/3/1 |
| KT734825 | TE6685/2-13 | 2063 | Italy | Canis lupus familiaris | 2013/3/1 |
| KT734822 | TE7482-13 | 2063 | Italy | Canis lupus familiaris | 2013/4/1 |
| KT734813 | AZ2972-13 | 2063 | Italy | Canis lupus familiaris | 2013/5/1 |
| KP260927 | VS7100005 | 2063 | United Kingdom | Vulpes vulpes | 2013/5/17 |
| KJ530972 | Bari/411-13 | 2063 | Italy | Canis lupus familiaris | 2013/6/1 |
| KP260926 | VS7100003 | 2063 | United Kingdom | Vulpes vulpes | 2013/6/3 |
| KP260925 | VS7100001 | 2063 | United Kingdom | Vulpes vulpes | 2013/6/10 |
| KT734815 | AZ4133/1-13 | 2063 | Italy | Canis lupus familiaris | 2013/7/1 |
| KT734823 | PE8575/1-13 | 2063 | Italy | Canis lupus familiaris | 2013/7/1 |
| KT734826 | PE8575/2-13 | 2063 | Italy | Canis lupus familiaris | 2013/7/1 |
| KT734827 | AZ4133/2-13 | 2063 | Italy | Canis lupus italicus | 2013/7/1 |
| KF887949 | Ha13 | 2063 | Germany | Canis lupus familiaris | 2013/7/22 |
| KT734816 | AZ4438-13 | 2063 | Italy | Canis lupus familiaris | 2013/9/1 |
| KT734819 | AZ5586-13 | 2063 | Italy | Canis lupus familiaris | 2013/11/1 |
| KT283604 | FUBerlin-JRS | 2063 | Germany | Canis lupus familiaris | 2014/6/1 |
| KT734817 | AZ5212/1-14 | 2063 | Italy | Canis lupus familiaris | 2014/10/1 |
| KT734818 | AZ5212/2-14 | 2063 | Italy | Canis lupus familiaris | 2014/10/1 |
| KT734828 | CB6293/2-14 | 2063 | Italy | Canis lupus | 2014/11/1 |
| KT734812 | CB6293/1-14 | 2063 | Italy | Canis lupus familiaris | 2014/11/1 |
| MF457592 | OH19098-1 | 2063 | USA | Canis lupus familiaris | 2015/8/21 |
| MG279135 | 201 | 2063 | China | Canis lupus familiaris | 2016/3/1 |
| MK033608 | UBA-Baires | 2063 | Argentina | Canis lupus | 2016/4/1 |
| MF797786 | XF16 | 2063 | China | Canis lupus familiaris | 2016/4/1 |
| MK731981 | C24 | 2063 | China | Canis lupus familiaris | 2016/4/1 |
| MK731982 | K1 | 2063 | China | Canis lupus familiaris | 2016/4/1 |
| MK944079 | C79 | 2063 | China | Canis lupus familiaris | 2016/4/1 |
| MG266899 | CD17/2016 | 2063 | China | Canis lupus familiaris | 2016/10/31 |
| MK944080 | C85 | 2063 | China | Canis lupus familiaris | 2018/8/1 |
| MT424579 | HENAN74 | 2063 | China | Canis lupus familiaris | 2019/1/1 |
| ON922899 | 212 | 2063 | China | Canis lupus familiaris | 2019/4/1 |
| OK625288 | IRN/2019/Dog/292 | 2063 | Iran | Canis lupus familiaris | 2019/4/1 |
| OK625289 | IRN/2019/Dog/298 | 2063 | Iran | Canis lupus familiaris | 2019/4/1 |
| NC_020904 | UCD1-1698 | 2063 | USA | Canis lupus familiaris | 2019/4/1 |
| OK625290 | IRN/2019/Dog/308 | 2063 | Iran | Canis lupus familiaris | 2019/8/1 |
| OK625291 | IRN/2019/Dog/496 | 2063 | Iran | Canis lupus familiaris | 2019/8/1 |
| OK625292 | IRN/2019/Dog/497 | 2063 | Iran | Canis lupus familiaris | 2019/8/1 |
| MN863535 | AH-1/2019 | 2063 | China | Canis lupus familiaris | 2019/10/1 |
| MN863536 | AH-2/2019 | 2063 | China | Canis lupus familiaris | 2019/10/1 |
| MN128702 | NC21 | 2063 | China | Canis lupus familiaris | 2019/10/1 |
| MZ826142 | WD-R009/TH2020 | 2063 | Thailand | Canis lupus familiaris | 2020/1/9 |
| MZ826143 | WD-R010/TH2020 | 2063 | Thailand | Canis lupus familiaris | 2020/1/9 |
| MZ826147 | WD-H026/TH2020 | 2063 | Thailand | Canis lupus familiaris | 2020/1/29 |
| MZ826144 | WD-R025/TH2020 | 2063 | Thailand | Canis lupus familiaris | 2020/3/20 |
| MZ826145 | WD-R081/TH2020 | 2063 | Thailand | Canis lupus familiaris | 2020/3/25 |
| MZ826146 | WD-R103/TH2020 | 2063 | Thailand | Canis lupus familiaris | 2020/3/25 |
| ON922898 | 32 | 2063 | China | Canis lupus | 2020/5/1 |
| MZ826148 | WD-H070/TH2020 | 2063 | Thailand | Canis lupus familiaris | 2020/6/17 |
| OK625293 | IRN/2019/Dog/498 | 2063 | Iran | Canis lupus familiaris | 2020/8/1 |
| ON922900 | 284 | 2063 | China | Canis lupus familiaris | 2020/10/1 |
| OQ627373 | CD0032 | 2063 | China | Canis lupus familiaris | 2022/10/12 |
| OQ910496 | CanineCV SC02 | 2063 | China | Canis lupus familiaris | 2022/10/23 |
| OQ910497 | CanineCV SC03 | 2063 | China | Canis lupus familiaris | 2022/10/23 |
| OQ910498 | CanineCV SC11 | 2062 | China | Canis lupus familiaris | 2022/10/23 |
| OQ910499 | CanineCV SC12 | 2063 | China | Canis lupus familiaris | 2022/10/23 |
| OQ910500 | CanineCV SC14 | 2063 | China | Canis lupus familiaris | 2022/10/23 |
| OQ910501 | CanineCV SC32 | 2063 | China | Canis lupus familiaris | 2022/10/23 |
| OQ910502 | CanineCV SC33 | 2062 | China | Canis lupus familiaris | 2022/10/23 |
| OQ910503 | CanineCV SC48 | 2064 | China | Canis lupus familiaris | 2022/10/23 |
| OQ910504 | CanineCV SC49 | 2063 | China | Canis lupus familiaris | 2022/10/23 |
| OQ910506 | CanineCV SC63 | 2063 | China | Canis lupus familiaris | 2022/10/23 |
| OQ910507 | CanineCV SC64 | 2063 | China | Canis lupus familiaris | 2022/10/23 |
| ON922902 | 352 | 2063 | China | Vulpes vulpes | 2022 |
| ON922903 | 44 | 2063 | China | Vulpes vulpes | 2022 |

**Table A4.** **Spatiotemporal spread of the Canine circoviruses in each genotype.**

| **Continent** | **Country** | **1996** | **1997** | **1999** | **2009** | **2010** | **2011** | **2012** | **2013** | **2014** | **2015** | **2016** | **2017** | **2018** | **2019** | **2022** |
| --- | --- | --- | --- | --- | --- | --- | --- | --- | --- | --- | --- | --- | --- | --- | --- | --- |
| Asia | China |  |  |  |  |  |  |  |  | CanineCV-2 CanineCV-3 | CanineCV-2 CanineCV-3 CanineCV-4 | CanineCV-1 CanineCV-3 CanineCV-4 | CanineCV-2 | CanineCV-2 CanineCV-4 | CanineCV-2 CanineCV-3 CanineCV-4 |  |
|  | Thailand |  |  |  |  |  |  |  |  |  |  | CanineCV-4 |  |  |  |  |
|  | VietNam |  |  |  |  |  |  |  |  |  |  |  | CanineCV-1 CanineCV-4 | CanineCV-1 CanineCV-4 |  |  |
|  | Iran |  |  |  |  |  |  |  |  |  |  |  |  |  | CanineCV-6 |  |
| Europe | Norway | CanineCV-5 | CanineCV-5 | CanineCV-5 |  |  |  |  |  | CanineCV-5 | CanineCV-5 |  | CanineCV-5 |  |  |  |
|  | UK |  |  |  |  |  |  |  | CanineCV-5 |  |  |  |  |  |  |  |
|  | Italy |  |  |  | CanineCV-1 CanineCV-5 | CanineCV-1 | CanineCV-4 |  | CanineCV-1 | CanineCV-1 |  | CanineCV-1 | CanineCV-1 CanineCV-5 | CanineCV-1 |  | CanineCV-1 |
|  | Croatia |  |  |  |  |  |  |  |  | CanineCV-5 |  |  |  |  |  |  |
|  | Germany |  |  |  |  |  |  |  | CanineCV-1 | CanineCV-1 |  |  |  |  |  |  |
| North America | USA |  |  |  |  |  | CanineCV-1 CanineCV-5 | CanineCV-1 |  |  | CanineCV-1 |  |  |  |  |  |
| South America | Argentina |  |  |  |  |  |  |  |  |  |  | CanineCV-1 |  |  |  |  |
|  | Brazil |  |  |  |  |  |  |  | CanineCV-1 |  |  |  |  |  |  |  |
|  | Colombia |  |  |  |  |  |  |  |  |  |  |  |  | CanineCV-1 |  |  |

**Table A5. Amino acid mutation site selected positive for Rep protein of canine circovirus (CanineCV).**

| **Group** | **Strain** | **Rep (Site)** | | | | | |
| --- | --- | --- | --- | --- | --- | --- | --- |
|  |  | **10** | **16** | **140** | **149** | **164** | **269** |
| **CanineCV 1** | **CanineCV 1 (major)** | **V** | **G** | **A** | **F** | **T** | **G** |
| **CanineCV 1** | **ON922896/canine/China** | **V** | **S** | **A** | **F** | **T** | **V** |
| **CanineCV 1** | **MT063075/canine/China** | **V** | **S** | **G** | **Y** | **T** | **G** |
| **CanineCV 1** | **MT063073/canine/China** | **V** | **S** | **A** | **Y** | **T** | **G** |
| **CanineCV 1** | **OM136006/canine/China** | **V** | **G** | **A** | **Y** | **T** | **G** |
| **CanineCV 1** | **MT063072/canine/China** | **V** | **G** | **A** | **Y** | **T** | **G** |
| **CanineCV 1** | **MT063074/canine/China** | **V** | **G** | **A** | **Y** | **T** | **G** |
| **CanineCV 1** | **MZ826148/canine/Thailand** | **V** | **G** | **A** | **F** | **T** | **A** |
| **CanineCV 1** | **MZ826147/canine/Thailand** | **V** | **G** | **A** | **F** | **T** | **V** |
| **CanineCV 1** | **ON922896/canine/China** | **V** | **G** | **A** | **F** | **T** | **A** |
| **CanineCV 2** | **CanineCV 2 (major)** | **V** | **G** | **A** | **F** | **T** | **G** |
| **CanineCV 2** | **MG279124/China** | **S** | **G** | **S** | **F** | **T** | **G** |
| **CanineCV 2** | **KY388499/canine/China** | **D** | **G** | **S** | **F** | **T** | **G** |
| **CanineCV 2** | **KY388497/canine/China** | **G** | **G** | **S** | **F** | **A** | **G** |
| **CanineCV 2** | **KY388487/canine/China** | **V** | **G** | **S** | **F** | **T** | **G** |
| **CanineCV 2** | **KY388502/canine/China** | **G** | **G** | **S** | **F** | **A** | **G** |
| **CanineCV 2** | **KY388484/canine/China** | **G** | **G** | **S** | **F** | **A** | **G** |
| **CanineCV 2** | **KY388500/canine/China** | **G** | **G** | **S** | **F** | **A** | **G** |
| **CanineCV 2** | **KY388492/canine/China** | **G** | **G** | **S** | **F** | **A** | **G** |
| **CanineCV 2** | **KY388501/canine/China** | **V** | **G** | **A** | **F** | **N** | **G** |
| **CanineCV 2** | **MG279136/China** | **G** | **G** | **A** | **F** | **T** | **A** |
| **CanineCV 2** | **MG279132/China** | **G** | **G** | **A** | **F** | **T** | **A** |
| **CanineCV 2** | **MG279138/China** | **G** | **G** | **A** | **F** | **T** | **A** |
| **CanineCV 3** | **CanineCV 3 (major)** | **G** | **G** | **A** | **Y** | **T** | **G** |
| **CanineCV 3** | **MN863537/canine/China** | **V** | **G** | **A** | **Y** | **T** | **G** |
| **CanineCV 3** | **MT063078/canine/China** | **V** | **G** | **A** | **Y** | **T** | **G** |
| **CanineCV 3** | **MT063079/canine/China** | **V** | **G** | **A** | **Y** | **T** | **G** |
| **CanineCV 3** | **MT063080/canine/China** | **V** | **G** | **A** | **Y** | **T** | **G** |
| **CanineCV 4** | **CanineCV 4 (major)** | **G** | **G** | **S** | **Y** | **T** | **S** |
| **CanineCV 4** | **MT740200/canine/Viet_Nam** | **V** | **G** | **S** | **Y** | **T** | **P** |
| **CanineCV 4** | **MT740201/canine/Viet_Nam** | **V** | **G** | **S** | **Y** | **T** | **P** |
| **CanineCV 4** | **MK033608/canine/Argentina** | **V** | **G** | **S** | **Y** | **T** | **P** |
| **CanineCV 4** | **KT734825/canine/Italy** | **V** | **G** | **S** | **Y** | **T** | **S** |
| **CanineCV 4** | **MT740198/canine/Viet_Nam** | **V** | **G** | **S** | **Y** | **T** | **P** |
| **CanineCV 4** | **OQ262878/canine/Namibia** | **V** | **G** | **S** | **Y** | **T** | **P** |
| **CanineCV 4** | **OQ262865/canine/Namibia** | **D** | **G** | **S** | **Y** | **T** | **P** |
| **CanineCV 4** | **OQ262885/canine/Namibia** | **V** | **G** | **S** | **Y** | **T** | **P** |
| **CanineCV 4** | **OQ262883/canine/Namibia** | **V** | **G** | **S** | **Y** | **T** | **P** |
| **CanineCV 4** | **MK424788/canine/Brazil** | **G** | **C** | **S** | **Y** | **T** | **A** |
| **CanineCV 4** | **MT193163/canine/Italy** | **G** | **G** | **S** | **F** | **T** | **P** |
| **CanineCV 4** | **ON922900/canine/China** | **G** | **G** | **S** | **F** | **T** | **P** |
| **CanineCV 4** | **MT193165/canine/Italy** | **G** | **G** | **S** | **Y** | **A** | **P** |
| **CanineCV 4** | **KT734821/canine/Italy** | **G** | **G** | **S** | **Y** | **A** | **S** |
| **CanineCV 4** | **KF887949/canine/Germany** | **G** | **G** | **S** | **Y** | **A** | **P** |
| **CanineCV 4** | **KT734825/canine/Italy** | **G** | **G** | **S** | **Y** | **A** | **S** |
| **CanineCV 4** | **MT193164/canine/Italy** | **G** | **G** | **S** | **Y** | **T** | **G** |
| **CanineCV 4** | **JQ821392/canine/USA** | **G** | **G** | **S** | **Y** | **T** | **G** |
| **CanineCV 5** | **CanineCV 5 (major)** | **V** | **G** | **A** | **F** | **A** | **G** |
| **CanineCV 5** | **MT180080/red_fox/Norway** | **V** | **R** | **A** | **F** | **A** | **G** |
| **CanineCV 5** | **ON418897/fox/Canada** | **V** | **R** | **A** | **F** | **A** | **G** |
| **CanineCV 5** | **ON418902/fox/Canada** | **V** | **R** | **A** | **F** | **A** | **G** |
| **CanineCV 5** | **MT180079/red_fox/Norway** | **V** | **G** | **G** | **F** | **A** | **G** |
| **CanineCV 5** | **MT180082/red_fox/Norway** | **V** | **G** | **G** | **F** | **T** | **G** |
| **CanineCV 5** | **ON418893/fox/Canada** | **V** | **G** | **A** | **F** | **T** | **G** |
| **CanineCV 5** | **ON418895/fox/Canada** | **V** | **G** | **A** | **F** | **T** | **G** |
| **CanineCV 5** | **ON418898/fox/Canada** | **V** | **G** | **A** | **F** | **T** | **G** |
| **CanineCV 5** | **ON418900/fox/Canada** | **V** | **G** | **A** | **F** | **T** | **G** |
| **CanineCV 5** | **ON418902/fox/Canada** | **V** | **G** | **A** | **F** | **T** | **G** |
| **CanineCV 5** | **ON418903/fox/Canada** | **V** | **G** | **A** | **F** | **T** | **G** |
| **CanineCV 5** | **ON418904/fox/Canada** | **V** | **G** | **A** | **F** | **T** | **G** |
| **CanineCV 5** | **ON418901/fox/Canada** | **V** | **G** | **A** | **F** | **S** | **P** |
| **CanineCV 5** | **ON418896/fox/Canada** | **V** | **G** | **A** | **F** | **A** | **A** |
| **CanineCV 5** | **ON418899/fox/Canada** | **V** | **G** | **A** | **F** | **A** | **P** |
| **CanineCV 5** | **MZ407653/fox/Italy** | **V** | **G** | **A** | **F** | **A** | **C** |
| **CanineCV 6** | **CanineCV 6 (major)** | **G** | **G** | **A** | **F** | **T** | **G** |
| **CanineCV 6** | **OK625289/canine/Iran** | **A** | **G** | **A** | **F** | **T** | **G** |
| **CanineCV 6** | **OK625291/canine/Iran** | **A** | **G** | **A** | **F** | **T** | **G** |
| **CanineCV 6** | **OK625293/canine/Iran** | **A** | **G** | **A** | **F** | **T** | **G** |
| **CanineCV 6** | **OK625290/canine/Iran** | **V** | **G** | **G** | **F** | **T** | **C** |
